# Characterization of Olive Oil Phenolic Extracts and Their Effects on the Aggregation of the Alzheimer’s Amyloid-β Peptide and Tau

**DOI:** 10.1021/acsomega.4c01281

**Published:** 2024-07-17

**Authors:** Bakri Alaziqi, Liam Beckitt, David J. Townsend, Jasmine Morgan, Rebecca Price, Alana Maerivoet, Jillian Madine, David Rochester, Geoffrey Akien, David A. Middleton

**Affiliations:** † Department of Chemistry, Lancaster University, Lancaster LA1 4YB, United Kingdom; ‡ Department of Chemistry, University College in Al-Qunfudah, Umm Al-Qura University, Makkah Al-Mukarramah 1109, Saudi Arabia; § Department of Biology, Edge Hill University, Ormskirk L39 4QP, United Kingdom; ∥ Department of Biochemistry, Cell and Systems Biology, Institute of Systems, Molecular and Integrative Biology, University of Liverpool, Liverpool L69 7ZB, United Kingdom

## Abstract

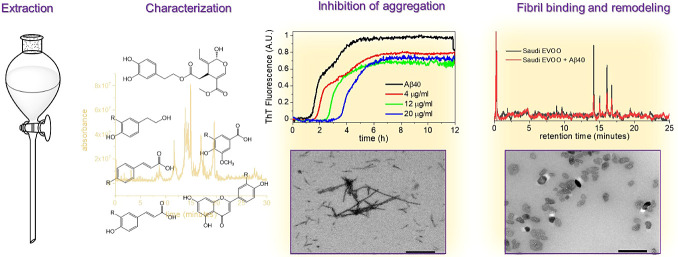

The dietary consumption of extra virgin olive oil (EVOO) is believed to slow the progression of Alzheimer’s disease (AD) symptoms. Its protective mechanisms are unclear, but specific EVOO phenolic compounds can individually impede the aggregation of amyloid-β (Aβ) peptides and the microtubule-associated protein tau, two important pathological manifestations of AD. It is unknown, however, whether the numerous and variable phenolic compounds that are consumed in dietary EVOO can collectively alter tau and Aβ aggregation as effectively as the individual compounds. The activity of these complex mixtures against Aβ and tau may be moderated by competition between active and nonactive phenolic components and by extensive derivatizations and isomerization. Here, phenolic mixtures extracted from two different EVOO sources are characterized and tested for how they modulate the aggregation of Aβ40 peptide and tau peptides *in vitro*. The chromatographic and NMR analysis of Greek and Saudi Arabian EVOO phenolic extracts reveals that they have different concentration profiles, and over 30 compounds are identified. Thioflavin T fluorescence and circular dichroism measurements show that relatively low concentrations (<20 μg/mL) of the Greek and Saudi extracts reduce the rate of Aβ40 aggregation and fibril mass, despite the extracts having different phenolic profiles. By contrast, the Greek extract reduces the rate of tau aggregation only at very high phenolic concentrations (>100 μg/mL). Most compounds in the extracts bind to preformed Aβ40 fibrils and release soluble Aβ oligomers that are mildly toxic to SH-SY5Y cells. Much higher (500 μg/mL) extract concentrations are required to remodel tau filaments into oligomers, and a minimal binding of phenolic compounds to the preformed filaments is observed. It is concluded that EVOO extracts having different phenol profiles are similarly capable of modulating Aβ40 aggregation and fibril morphology *in vitro* at relatively low concentrations but are less efficient at modulating tau aggregation. Over 2 M tonnes of EVOO are consumed globally each year as part of the Mediterranean diet, and the results here provide motivation for further clinical interrogation of the antiaggregation properties of EVOO as a potential protective mechanism against AD.

## Introduction

The consumption of olive oil in the so-called Mediterranean diet has been linked to a decreased incidence of cardiovascular disease, diabetes, some cancers, and neurodegenerative disorders.^1−4^ In common with many plant-derived dietary substances, unprocessed extra virgin olive oil (EVOO) contains various phenolic compounds, which are known to have antioxidant and anti-inflammatory properties.^5−10^ Several natural phenolic compounds have been investigated for their ability to cross the blood-brain barrier (BBB) and potentially delay the onset of the pathological hallmarks of Alzheimer’s disease (AD). Their principal therapeutic mechanism in this regard appears to be the scavenging of free radicals and the prevention of neuronal oxidative damage. However, data obtained *in vitro* suggest that individual phenolic compounds may also reduce or delay the deposition of protein aggregates that are pathological signatures of AD.^11,12^

AD is associated with the 39–42 residue amyloid-β (Aβ) peptides that assemble into β-sheet-rich, insoluble amyloid fibrils and accumulate within heterogeneous plaques in the extracellular spaces of brain tissue.^13^ Amyloid fibrils are insoluble nanoscale fibrous structures, typically 10 nm in diameter and micrometers long, that can be identified by the characteristic cross-β pattern seen by X-ray fiber diffraction and by the green birefringence displayed upon binding to Congo red.^14^ Fibril formation occurs via transitory oligomeric species that are toxic to neuron synapses and disrupt cell membranes.^15−20^ A further characteristic of AD is the hyperphosphorylation of microtubule-associated protein tau (MAPT, or tau; UniProtKB P10636), by glycogen synthase kinase enzymes, which triggers aggregation into neurofibrillary tangles associated with neurodegeneration, and this is thought to succeed the Aβ aggregation and related inflammatory response.^21−24^ Antiaggregation drugs that impede the formation of amyloid fibrils and filaments *in vivo* continue to be investigated for the treatment of AD and other amyloid diseases.^25−27^

Polyphenols consumed in olive oil as part of a normal diet are interesting from a therapeutic perspective because certain molecules of this class have been shown to reduce the rate of Aβ and tau aggregation and destabilize fibrils.^28−30^ Olive oil is the source of oleuropein, a catechol-containing compound that, in its aglycone form, inhibits the aggregation of Aβ, tau, and other proteins *in vitro* and can ameliorate amyloid pathologies *in vivo*.^31−36^ Oleuropein can exist in the aglycone form or as an ester of elenolic acid and hydroxytyrosol, linked to glucose via a glycosidic bond.^31−34,37^ Oleuropein and its metabolic products hydroxytyrosol and tyrosol are often the most abundant phenolic compounds in EVOO,^38,39^ and the amyloid-inhibiting and clearing properties of the individual compounds have been studied in detail.^37,39−42^ However, EVOO is also a rich source of many other types of phenolic compounds, including flavonoids,^40^ lignans,^41,42^ hydroxybenzoic acids,^43,44^ and phenolic acids.^45−47^ Several compounds from these classes, including hydroxycinnamic (coumaric) acids,^48^ ferulic acid,^49,50^ and the flavonoids quercetin^51^ and apigenin,^52^ have been shown individually to inhibit Aβ and/or tau aggregation, disrupt fibrils, and relieve AD-like pathologies in animal models.^53^ Another major olive oil polyphenol, oleocanthal, modulates tau fibrillization^54^ and enhances the clearance of Aβ fibrils from the brain.^55^

Studies of the antiaggregation properties of EVOO polyphenols *in vitro* have focused exclusively on the effects of individual compounds, such as oleuropein aglycone. While such studies provide important mechanistic information, the compounds studied to date represent a small fraction of the diverse polyphenols that are consumed with EVOO in the Mediterranean diet. Oleuropein, for one, occurs in EVOO as the aglycone, glucosides, and in other derivative forms and also as different isomers (*e.g.*, aldehydic). Other phenolic compounds exhibit similar structural diversification around the parent compounds,^56^ and it is not known how the vast majority of them affect amyloid aggregates. Further, the concentration profile of EVOO phenols and derivatives is highly sample-dependent and varies according to olive growing conditions, harvest time, and processing.^57^ The amyloid-modifying properties of different EVOO samples may therefore be similarly variable in samples of different provenance. Moreover, competition between the phenolic components of EVOO mixtures for binding to amyloid may reduce the antiaggregation effects as compared to the individual compounds, particularly if nonactive compounds compete with nonactive compounds. A further consideration is the extent to which EVOO phenolic mixtures are absorbed and metabolized *in vivo* compared to individual phenolic compounds. It is therefore far from certain, without experimental verification, whether the amyloid-modifying properties of phenolic mixtures in EVOO are as potent as those of the individual EVOO compounds.

To address some of these uncertainties, we report the first analysis of the antiaggregation properties of phenolic mixtures from EVOO and compare the effects of polyphenols extracted from two distinct EVOO sources (Greece and Saudi Arabia). The content of the extracts is analyzed and evaluated for their effects on the aggregation of Aβ40 (residues 672–711 of the amyloid precursor protein; UniProtKB P05067) and a recombinant tau fragment Δtau187, comprising residues 255–441 of the C-terminal microtubule-binding domain.^58^ Over 30 compounds from the extract were identified by LC-MS and HPLC analyses, including oleuropein derivatives, simple phenolic acids, and flavonoids. It is shown that the extract mixtures from the two sources have a much greater effect on Aβ40 aggregation and destabilization of Aβ40 fibrils than they do on tau around equimolar concentrations with respect to the proteins.

## Materials and Methods

### Aβ40 Expression

Human Aβ40 comprising the amyloidogenic 1–40 residues with an additional N-terminal methionine residue was expressed and purified as previously described.^59^ All experiments involving Aβ40 were conducted in 25 mM phosphate, 0.1% NaN_3_, pH 7.4.

### Tau Expression

The tau construct used in this work comprises residues 255–441 of human tau from cDNA clone htau46, and the protein was expressed and purified as previously described.^60^ This isoform consists of the four microtubule-binding (MTB) repeat units (tau 4R) but with the aggregation impeding N-terminus removed, leaving the second and third MTB with the highly amyloidogenic sequences VQIINK and VQIVYK, respectively.^61^ All experiments involving tau were conducted in 30 mM Tris, 1 mM DTT, pH 7.5.

### Polyphenol Extraction from EVOO

The extra virgin olive oils used in this study are commercially available from Greece (Yannis Fresh Greek Early Harvest Extra Virgin Olive Oil cold extraction) and from Saudi Arabia (Buseita Al Jouf Early Harvest Extra Virgin Olive Oil first and cold press). Bottles were covered with foil and stored away from light at 4 °C. The polyphenol extraction protocol was derived from previously reported methods.^5^ Briefly, 10 g of olive oil was dissolved in 50 mL hexane, and the solution was sonicated at 20 μm for 5 min. The solution was then loaded into the separating funnel and shaken for 2 min before extracting with 20 mL of methanol/water (60:40, v/v) three times to extract. The methanolic phases containing the polar polyphenol compounds were collected, washed twice with hexane, and refrigerated for 24 h. The methanol was removed at 40 °C under reduced pressure, prior to lyophilization at −70 °C and 0.0026 mbar pressure for 24 h. The solid was weighed and resuspended in 50:50 methanol/water to a final concentration of 10 mg/mL.

### Solution-State NMR Analysis of EVOO Extracts

Polyphenol extract samples were prepared for solution-state NMR by dissolving ca. 40 mg in 0.6 mL DMSO-d6, and the acidity was carefully adjusted by the stepwise addition of DMSO-d6 stock solutions of trifluoroacetic acid (TFA) or triethylamine and monitoring the line width of the hydroxyl peaks in 1D proton spectra. It appears that excessive amounts of triethylammonium trifluoroacetate also catalyze proton transfer and give unwanted line-broadening, and the optimal acidity was in quite a narrow concentration range so it was rather easy to overshoot. Typically this amounted to the cumulative addition of 10–20 μL of a 100 mM TFA stock and polyphenol hydroxyl line widths on the order of 1–2 Hz. Magnitude-mode DQF-COSY spectra were acquired with gradients using the Bruker library sequence cosygpmfppqf, an offset of 5.5 ppm, and 4 kHz spectral widths in both the direct and indirect dimensions. 256 t_1_-increments were acquired with two transients per increment.

### LC-MS Analysis of EVOO Extracts

The LC-MS analysis of the extract mixtures was conducted using a Shimadzu LCMS-IT-TOF instrument fronted by a NexeraX2 UHPLC instrument consisting of a DGU-20A5R degassing unit, two LC-30AD LC pumps, a SIL-30AC autosampler, and a CTO-20AC column oven. Separation achieved using the same Shim-pack XR-ODS 2.2 μm (3.0 × 50 mm) column optimum separation was ensured using a binary mobile-phase gradient at a 1 mL/min flow rate. The column temperature was maintained at 40 °C with a 20 μL injection volume. Further, the solvents included 0.1% formic acid in ultrapure water (buffer A) or acetonitrile (buffer B). Following was the gradient elution program: 0–3 min, 5% B; 3–25 min, 40% B; 25–26 min, 40% B; 26–27 min, 50% B; 27–27.10 min, 5% B; and 27.10–32 min, 5% B. Data acquisition was performed in positive and negative ionization with polarity switching. Both positive and negative acquisitions range from 100 to 700 *m*/*z* with ion accumulation at 40 ms. Shimadzu LC-MS solution software was used to analyze the data. The predicted *m*/*z* value of [M + H]^+^ ions and [M – H]^−^ ions in positive and negative ionization scan modes was used to calculate sample peak areas.

### HPLC Analysis of EVOO Extracts

Separation of the EVOO extracts was achieved on a NexeraX2 UHPLC (Shimadzu) system at 40 °C with a mobile phase consisting of 0.1% formic acid in ultrapure water (solvent A) mixed in various proportions with acetonitrile (solvent B). The solid phase consisted of a Shim-pack XR-ODS 2.2 μm (3.0 × 50 mm) column. From a 1 mg/mL olive oil extract, stock solution was loaded 10 μL and the system was run at a flow rate of 1 mL/min, with a reverse-phase elution profile of 5% solvent B for 0–3 min, 40% solvent B for 3–26 min, 50% solvent B for 26–27 min, and 5% solvent B for 27–32 min. A diode array detector enabled the spectroscopic absorbance of each chromatographic peak to be measured from 240 to 400 nm.

Certain compounds could be identified and quantified by measuring the HPLC retention times and peak intensities of reference samples of known concentration. For these compounds, standard plots of concentration vs absorbance at 240, 275, or 340 nm were prepared in the linear range, and the gradients of the plots were used to determine the unknown concentrations of the extracted compounds from their peak intensities. Standard solutions (all from a 500 μM stock) were as follows: tyrosol (purity 98%), hydroxytyrosol (purity 98%), vanillic acid (purity 97%), syringic acid (purity 98%), cinnamic acid (purity 99%), ferulic acid (purity 99%), *p*-coumaric acid (purity 98%), caffeic acid (purity 98%), and oleuropein aglycone and glucoside (purity 98%), all purchased from Sigma-Aldrich. Luteolin (purity 97%), apigenin (purity 97%), and naringenin (purity 97%) were purchased from Alfa Aesar, and (+)-pinoresinol (purity 95%) was obtained from Cayman Chemical Cambridge Bioscience. Preparation of standard stock solutions of each polyphenol was achieved by dissolving the appropriate small amount of the pure solid reagent in 25 mL of methanol/water. Appropriate dilution of standard working solutions at various concentrations was prepared when needed. All working solutions were prepared in a 10 mL volumetric flask and stored in the dark at 4 °C. Before injection into the UHPLC and LC-MS, all solutions passed through a 0.22 μm filter.

### Analysis of Phenol Binding to Protein Aggregates

The binding of the extracts to the Aβ40 and tau aggregates was quantified by two methods: reverse HPLC and UV spectrophotometry. For both methods, aggregates of Aβ40 or tau (20 μM monomer equivalent) were first formed by incubation of the proteins in 500 μL phosphate buffer at 37 °C for 3 days. The insoluble fibrils were sedimented by benchtop centrifugation, and the top 480 μL of supernatant was removed. The remaining pellet was resuspended in 480 μL phosphate buffer (pH 7.4) containing 20 μg/mL EVOO extract, and homogenized. The samples were incubated with agitation at 37 °C for a further 24 h before centrifuging again. The supernatants were retained for analysis. Control samples of EVOO extracts were prepared by following the procedure above but omitting the fibrils.

The centrifuged solutions obtained with and without fibrils were analyzed by reverse-phase HPLC using the method described in the previous section. From each solution, 10 μL was injected into the column. For each measurable peak resolved in the HPLC chromatogram, the percentage of the corresponding compound bound to the fibrils was calculated from the intensity ratio 100(1–*I*_f_)/*I*_c_, where *I*_f_ is the peak intensity for the fibril-treated sample and *I*_c_ is the peak intensity for the control sample. Certain compounds could be identified using reference samples as described before. For UV spectrophotometric analysis of binding, all samples already prepared for HPLC binding were examined using UV–visible spectra in the range of 200–500 nm using a NanoDrop 2000/2000c instrument.

The same procedure and the HPLC method were used for resolving caffeic acid (20 μM) and naringenin (20 μM) to Aβ40 fibrils (20 μM monomer equivalent). After centrifugation, the supernatants were analyzed using the Bradford method and by SDS-PAGE to confirm that no protein species remained in the supernatant.

### Thioflavin T Fluorescence Analysis of Aggregation Kinetics

The kinetics of amyloid formation were monitored from the fluorescence emission at 482 nm of the amyloid-specific dye thioflavin T (ThT). Fluorescence is enhanced in the presence of amyloid and follows an approximately sigmoidal pattern representing the lag, growth, and maturation phases of protein aggregation. Aβ40 (20 μM) or tau with heparin (20 and 5 μM, respectively) were incubated in a total volume of 200 μL in the presence of 20 μM ThT, with the inclusion of various concentrations of EVOO extracts or 20 μM of each of the polyphenol reference compounds caffeic acid, *trans-*cinnamic acid, *p*-coumaric acid, ferulic acid, tyrosol, vanillic acid, luteolin, apigenin, and naringenin. Fluorescence measurements, with excitation at 450 nm and emission at 482 nm, were taken (*n* = 3 per sample group) on a Molecular Devices Flexstation 3 Microplate Reader (Molecular Devices), every 2 min for 50 h. The samples were continually shaken at 37 °C during the incubation.

### Analysis of Protein Aggregation by Circular Dichroism Spectroscopy

Amyloid beta (20 μM) was incubated at 37 °C alone or in the presence of EVOO extracts in a range of concentrations or 20 μM of each of the polyphenol compounds, and spectra were acquired immediately after preparation and again after incubation for 2 and 24 h. Spectra were recorded on a Chirascan Plus CD spectrometer between 180 and 260 nm with a bandwidth of 1 nm, using a path length of 0.1 mm. Background signals of buffer and the relevant compound were removed from the spectra.

### Visualization of Aggregate Morphology by Transmission Electron Microscopy

Aβ40 (20 μM) and tau with heparin (20 and 5 μM, respectively) were incubated alone, or in the presence of phenolic extracts or individual components for 3 days. Phenolic extracts were added to the protein at the start of incubation or after fibril formation. For measurements on Aβ40 treated with the extracts at the start of incubation, the final EVOO extract concentration was 20 μg/mL. For measurements on Aβ40 treated with the extracts after fibril formation, fibril pellets obtained by centrifugation were resuspended in 500 μL phosphate buffer containing the EVOO extract at 20, 72, or 740 μg/mL. These samples were incubated for a further 24 h, and the insoluble and soluble fractions were separated by centrifugation for visualization by TEM. A 10 μL suspension was spotted onto Formvar and carbon-coated copper grids. After 5 min, the excess liquid was removed via blotting. For negative staining, 10 μL of 2% phosphotungstic acid was spotted onto the loaded grids and left for 3 min before blotting the excess. Grids were viewed on a JEOL JEM-1010 or JEOL 1400 Flash transmission electron microscope and images that were representative of the entire grid were captured.

### Solid-State NMR of Aβ40 Aggregates

For solid-state NMR (SSNMR), 740 μg Greek EVOO extract was added to sedimented fibrils in a small volume of phosphate buffer and, after thorough mixing, the fibrils were incubated at 37 °C for a further 24 h. Residual liquid was removed by centrifugation, and the pellet was transferred to a 3.2 mm magic angle spinning (MAS) rotor. ^15^N cross-polarization (CP-MAS) SSNMR spectra of uniformly ^15^N ([U–^15^N]) Aβ40 fibrils in the absence and presence of Greek EVOO extract were acquired at a ^1^H Larmor frequency of 700.13 MHz on a Bruker Avance 700 spectrometer. Insoluble fibrils formed after 3 days of incubation at 37 °C were isolated by centrifugation. A proton-decoupled ^15^N CP-MAS NMR spectrum was obtained at 10 kHz MAS with the following parameters: excitation of ^1^H magnetization was achieved with a 3 μs π/2 pulse, followed by a 2 ms contact time during which a ramped proton field of 63 kHz was matched to a ^15^N field to achieve the Hartmann–Hahn condition. The signal was acquired with 63 kHz proton decoupling using the SPINAL-64 sequence. A ^1^H–^15^N refocused INEPT spectrum was obtained at the same MAS frequency with π/2 and π pulses of 3 and 6 μs at the ^1^H frequency and 4 and 8 μs at the ^15^N frequency, respectively, with interpulse delays of 1 ms. Spectra were recorded at ambient temperature.

### Cell Viability

The cell viability experiment was performed in tandem with TEM to assess whether the addition of EVOO extracts to insoluble fibrils released soluble, cytotoxic oligomers. SH-SY5Y cells were maintained in Dulbecco's Modified Eagle Medium (DMEM) with 10% fetal bovine serum (FBS), 1% penicillin–streptomycin, and 1% nonessential amino acids. Cells were added to 96-well plates at 8000 cells per well in 80 μL and incubated at 37 °C with 5% CO_2_ for 24 h. Aβ40 fibrils alone (20 μM) were formed after incubation at 37 °C for 2 days and then centrifuged on a benchtop instrument. For control samples, the pellets were isolated, homogenized with phosphate buffer (500 μL), and incubated at 37 °C for a further 24 h. The samples were centrifuged again, and the supernatant was taken for addition to the cells. For EVOO extract-treated samples, the Aβ40 pellet obtained after the first centrifugation step was treated with 72 μg/mL or 144 μg/mL Greek EVOO in 500 μL phosphate buffer and incubated at 37 °C for 24 h. The samples were centrifuged and the supernatants were isolated. The control and EVOO-treated supernatants (*n* = 6 per group) were added to the cells (in 20 μL buffer per well) and incubated for a further 48 h. After this time, 10 μL of Cell Counting Kit-8 (CCK-8, Stratech) was added to each well, and the absorbance was recorded at 450 nm/650 nm over 3 h. A 650 nm reference wavelength was used to correct for the addition of insoluble fibrillar material (as per CCK-8 dye instructions). Data were processed and analyzed in GraphPad to report % viability in comparison with live (buffer alone) and dead (1% triton final concentration) controls using one-way ANOVA with Tukey’s multiple comparison correction.

### Dynamic Light Scattering

Dynamic light scattering was conducted using a Malvern Panalytical Zetasizer Nano ZSP at room temperature. Twelve measurements were carried out in triplicate for each sample (Aβ40 fibrils alone and following the addition of 144 μg/mL of Greek EVOO for 24 h) and averaged profiles produced.

### Molecular Docking

Computer docking was performed of individual polyphenol compounds to fibrillar structural models of tau (PDB 6QJH)^53^ and Aβ (PDB 2LMQ).^54^ All docking simulations were conducted using Molsoft ICM-Pro 3.9-1a software. The PDB files were converted into ICM files with tightly bound water molecules remaining, and hydrogen, histidine, proline, glutamate, glycine, and cysteine residues were all optimized. Initially, binding pockets were identified using the ICM Pro Pocket Finder algorithm, with a tolerance setting of 3, and ordered by volume size. The ICM files were then prepared for docking with the initial ligand position left unchanged in its starting location. The docking simulations were initiated with a thoroughness of 10 and 3 conformations using the Chemical Table option, which was populated with chemical structures for each target compound from the ChEMBL database.

## Results

### Identification of EVOO Phenolic Compounds

Two EVOO extracts from different sources were prepared to analyze the differences in their phenolic profiles before testing their effects on Aβ40 and tau aggregation. Polyphenols were extracted from EVOO obtained from Greek and Saudi Arabian olive sources, using an established polar-phase extraction method with further optimization. Three different extraction techniques, liquid–liquid extraction (LLE) with funnel separation, LLE with centrifugation, and solid-phase extraction (SPE), were applied to isolate polyphenolic compounds from EVOOs, in order to determine the effectiveness of each technique and to identify the technique that extracts reproducibly the highest polyphenol yield. The hexane/methanol funnel separation method yielded the highest amount of extract (25.3 mg solid per 10 g EVOO) from both sources, and this method was used throughout. The two extracts are henceforth referred to by their country of origin (Greek and Saudi), but no significance is attributed to their geographical source above the many other (*e.g.*, harvesting and manufacturing) variables that can influence the final polyphenol composition of the products.

**Figure 1:**
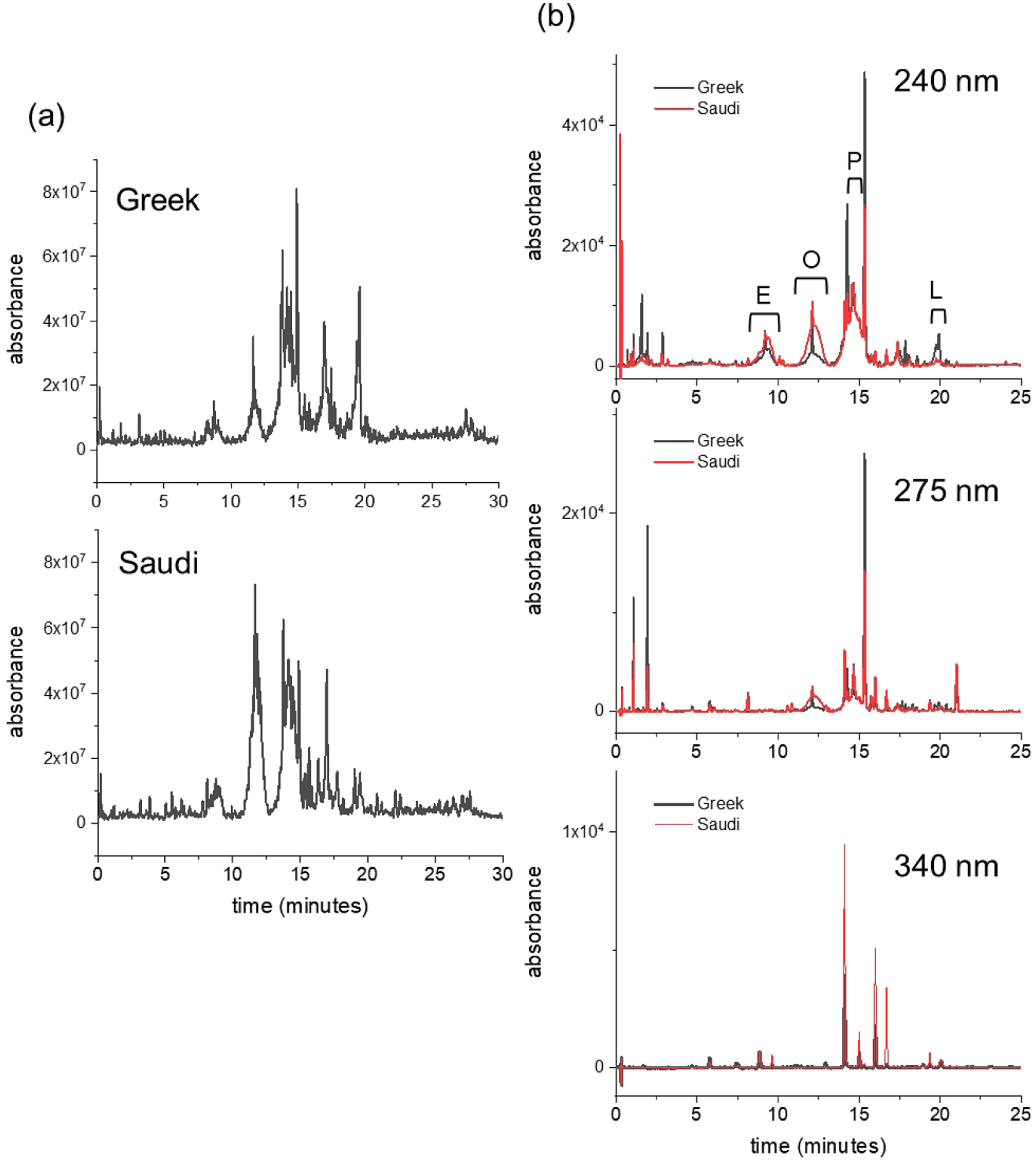
Chromatographic analysis of polyphenol extracts from two EVOO sources. (a) LC-MS chromatograms of the extracts from Greek EVOO (top) and from the Saudi EVOO (bottom). (b) Reverse-phase HPLC chromatogram at three wavelengths of the extract from the Greek EVOO sample (black) overlaid with the chromatogram of the Saudi extract (red). A stock solution of the extracts (10 mg/mL) in 50:50 v/v methanol/water was diluted to 1 mg/mL in water for the analysis. The polyphenol content and selected polyphenol concentrations of each extract are given in Tables 1 and 2. Broad peaks at 240 nm are assigned to E = elenolic acid derivatives; O = oleuropein derivatives; P = pinoresinol derivatives; L = ligstroside derivatives.

LC-MS was used in the first instance to identify the compounds present in the extract mixtures (Figure 1a). The individual MS profiles were scanned for the presence of polyphenol compound masses (H^+^, H^–^, and Na^+^) commonly detected in EVOO. These compounds and their empirical formulas are summarized in Table 1. LC-MS resolved 32 chromatographic peaks corresponding to 20 individual polyphenolic compounds and their isomers. Many of the previously reported EVOO polyphenol compounds were present in both mixtures, including oleuropein, elenolic acid, tyrosol, and hydroxytyrosol derivatives (including aglycones and glucosides) and derivatives of oleocanthal, which give early harvest EVOO its strong bitter taste. Phenolic acids (*e.g.*, caffeic acid, coumaric acid, and ferulic acid), dihydroxybenzoic acids (*e.g.*, vanillic acid), lignans, and flavonoids (*e.g.*, apigenin) were also detected. Oleuropein, tyrosol, and ferulic acid, apigenin, have previously been shown to inhibit Aβ aggregation and or disrupt fibrils *in vitro*. Virtually all the compounds detected were present in both Greek and Saudi samples.

**Table 1:** Summary of the Phenolic Compounds in the EVOO Extracts from Greek (G) and Saudi (S) Sources Identified by LC-MS

name	RT (min)	H (+)	H (−)	Na (+)	G	S
quinic acid	0.31		191.0567		Y	Y
hydroxytyrosol	1.20		153.0585		Y	Y
vanillic acid	3.19	169.0846			Y	Y
caffeic acid	3.64		179.0323		Y	Y
hydroxyelenolic acid (isomer 1)	6.91		257.0655		Y	N
hydroxyelenolic acid (isomer 2)	7.41		257.0664		Y	Y
hydroxyelenolic acid (isomer 3)	8.74	259.0777	257.0660		Y	Y
elenolic acid	9.00	243.0820	241.0722		Y	Y
hydroxyelenolic acid (isomer 4)	9.57		257.0657		Y	Y
hydroxydecarboxymethyl-oleuropein aglycone	11.63	337.1235	335.1113	359.1068	Y	Y
dihydroxyoleuropein aglycone (isomer 1)	11.70		409.1074		Y	N
hydroxytyrosol acetate	11.72		195.0677		Y	Y
dihydroxyoleuropein aglycone (isomer 2)	11.79		409.1077		Y	N
luteolin	13.74	287.0508	285.0407		Y	Y
decarboxymethyl-oleuropein aglycone	13.83	321.1300	319.1179	343.1103	Y	Y
pinoresinol (+)-	14.20		357.1105		Y	Y
oleocanthal*	14.26	305.1361	303.1238	327.1169	Y	Y
naringenin	14.91		271.0633		Y	Y
hydroxyoleuropein aglycone (isomer 1)	15.13	395.1309	393.1184	417.1399	Y	Y
hydroxyoleuropein aglycone (isomer 2)	15.48	395.1320	393.1174	417.1181	Y	Y
Name	RT (min)	H (+)	H (−)	Na (+)	G	S
apigenin	15.65	271.0562	269.0448		Y	Y
hydroxyoleuropein aglycone (isomer 3)	15.84	395.1319	393.1158	417.1237	Y	Y
tyrosol glucoside (salidroside)	16.32	301.0673	299.0551		N	Y
oleuropein aglycone (isomer 1)	16.95	379.1363	377.1230	401.1179	Y	Y
ligstroside aglycone (isomer 1)	17.24	363.1430	361.1302	385.1244	Y	Y
oleuropein aglycone (isomer 2)	17.44	379.1344	377.1230	401.1189	Y	Y
oleuropein aglycone (isomer 3)	17.70	379.1381	377.1230	401.1201	Y	Y
keto oleuropein aglycone	17.95	393.1196	391.1028		Y	N
ligstroside aglycone (isomer 2)	19.41	363.1389		385.1238	Y	Y
ligstroside aglycone (isomer 3)	19.59	363.1409		385.1208	Y	Y
ligstroside aglycone (isomer 4)	20.20	363.1439		385.1243	Y	N

A further, partially quantitative analysis of both mixtures was performed using reverse-phase HPLC with an elution gradient of up to 50% acetonitrile in water. UV–visible absorption spectra of the Greek and Saudi extracts exhibit 2 main bands with maxima at 240 and 275 nm and a “tail” from 300 to 400 nm, each differing in relative absorbance. The majority of compounds absorb at 240 nm, whereas conjugated molecules, such as some flavonoids, are more visible at wavelengths above 300 nm. The HPLC chromatogram is therefore shown at detection wavelengths of 240, 275, and 340 nm in Figure 1b, so as to visualize the majority of compounds in the extract. The HPLC chromatogram exhibits a distribution of sharp peaks as well as several broader peaks at retention times up to 25 min. From previous analyses,^38,62,63^ these broader peaks were here attributed putatively to various elenolic acid derivatives at 9.4 min, oleuropein derivatives at 12.3 min, (+)-pinoresinol derivatives at 14.6 min, and ligstrosides at 19.8 min. The HPLC profiles for the two mixtures at different wavelengths (Figure S1) revealed distinct differences in the relative proportions of the constituents. At 240 nm, most of the more prominent narrow peaks are higher for the Greek extract than for the Saudi extract, whereas the broader peaks at 9.4 and 12.3 min are larger in the chromatogram of the Saudi sample. At 340 nm, where conjugated aromatic compounds such as flavonoids are most strongly absorbing, the majority of the peaks are of higher absorbance in the Saudi extract. Hence, the Greek and Saudi extracts have somewhat distinct phenol concentration profiles.

A limited number of commercially available reference standards was used to assign some of the sharp peaks in the chromatograms and to determine the concentration of the corresponding compounds (Tables S1 and 2). Peaks and corresponding concentrations were determined for tyrosol and hydroxytyrosol, caffeic acid, oleuropein aglycone, and the flavonoids apigenin, luteolin, and naringenin. The Greek extract contains a higher proportion of oleuropein and its tyrosol metabolites than does the Saudi extract but has lower concentrations of the flavonoids luteolin and apigenin. The broader peak profiles were not assigned definitively. The majority of the peaks that differ between the two extracts remain unassigned, however. Together, the compound profiles identified by LC-MS and HPLC agree with previous analyses using elution gradients of up to 30%^64^ and 100%^38^ acetonitrile, and no major EVOO phenolic compounds were undetected as compared to the previous reports.

**Table 2:** Summary of the Phenolic Compounds from Greek and Saudi Extracts Identified and Quantified by HPLC^a^

polyphenolic compounds	retention time (min)	EVOO extract concentration (μg/g EVOO)	
		Greek	Saudi
hydroxytyrosol	1.0	15.70	8.52
tyrosol	1.9	23.11	6.92
vanillic acid	2.9	0.51	0.52
caffeic acid	3.0	0.07	0.09
*p*-coumaric acid	5.7	0.50	0.32
ferulic acid	7.4	0.10	0.18
oleuropein aglycone	13.0	10.42	3.55
luteolin	14.1	1.84	4.72
(+)-pinoresinol	14.6	10.78	14.53
naringenin	15.3	15.68	10.19
apigenin	16.0	1.05	3.76

a The wavelength at which the peak for each compound could be most reliably measured is also stated.

**Figure 2:**
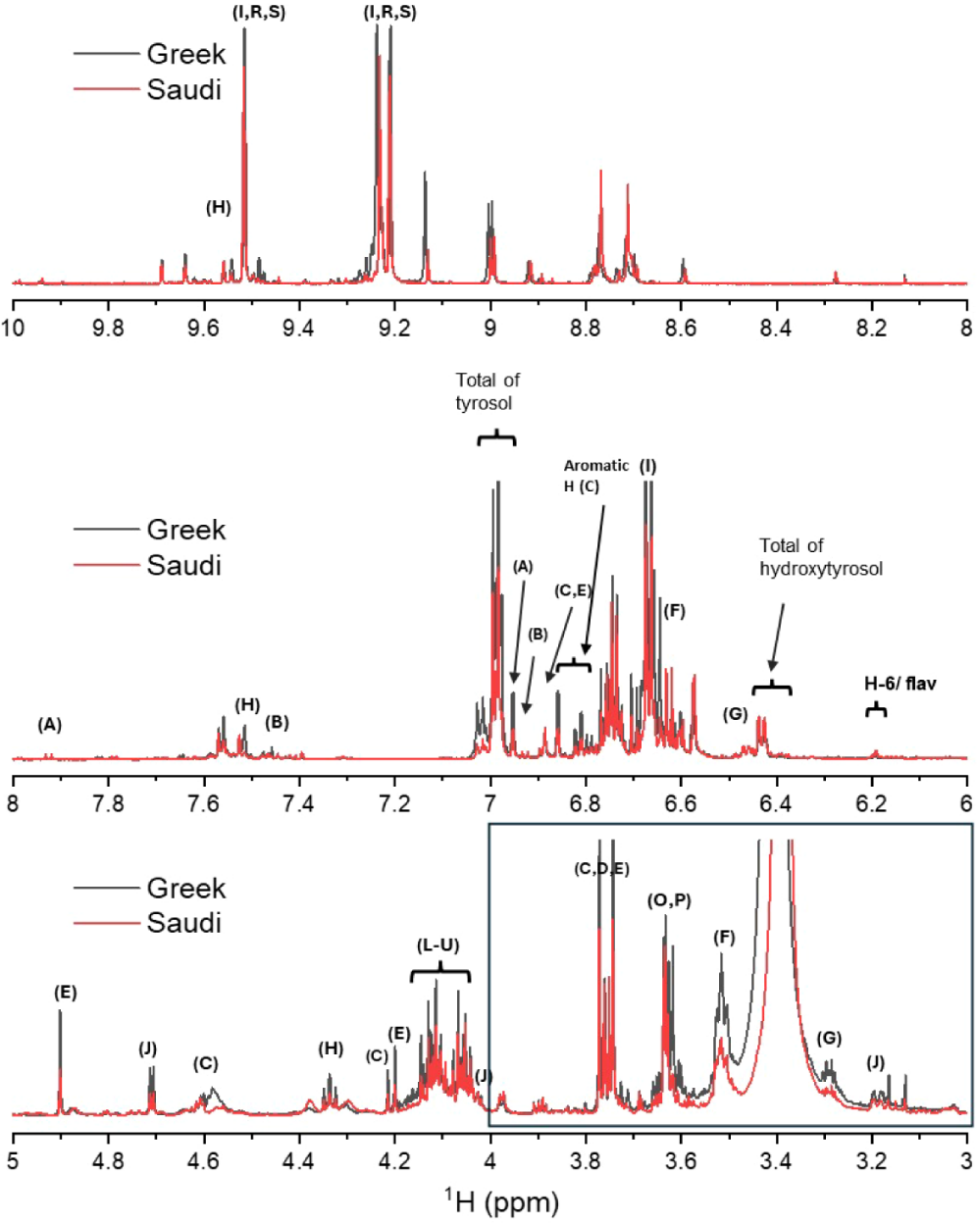
Solution-state ^1^H NMR spectra (700 MHz) of polyphenol extracts (in DMSO-*d*_6_) from Greek (black) and Saudi (red) EVOO samples. Top: low-field region showing resonances from aldehydic protons. Middle: aromatic region. Bottom: midfield region corresponding to the 2D COSY spectrum shown in Figure S2. The boxed region is where resonances from glucoside groups are expected. A: apigenin, B: luteolin, C: pinoresinol, D: syringaresinol, E: 1-acetoxypinoresinol, F: tyrosol, G: hydroxytyrosol, H: elenolic acid, I: oleocanthal, J–M: oleuropein and ligstroside glucosides and aglycones, N: dialdehydic form of oleuropein, O,P: aldehydic forms of oleuropein and ligstroside, Q–U: oleocanthal derivatives.

Additional analysis was performed using solution-state ^1^H NMR (Figure 2), to determine whether the mixture consisted of phenolic glucosides. It was not attempted to fully assign the spectra, but some peaks from specific compounds could be identified with reference to previous work. The region from 8 to 10 ppm contains many dispersed signals from aldehydic protons, which feature in oleuropein (dialdehydic form), oleocanthal, and elenolic acid among other compounds.^65,66^ The region from 3.3–5 ppm contains resonances from glucosyl groups, which were resolved to some extent in the 2D COSY spectrum (Figure S2).^65,66^ This indicates that certain phenols, such as oleuropein, pinoresinol, ligstroside, and others, exist in the *O*-glucoside forms, which may affect their ability to interact with Aβ40 and tau. The NMR spectra concur with the LC-MS HPLC profiles and indicate that most of the same compounds are present in the Greek and Saudi extracts but in different relative proportions.

### Effect of Individual EVOO Phenolics on Aβ40 Aggregation Kinetics

The chromatographic and NMR analysis identified compounds of the flavonoid, phenolacrylic acid, hydroxybenzoic acid, and secoiridoid classes, members of which are known to inhibit Aβ aggregation *in vitro*.^67^ We compared how representatives of these classes from EVOO affect Aβ40 and tau aggregation and bind to preformed Aβ40 and tau fibrils. Compounds were tested alone and when mixed together, to assess whether competition between phenolics in a mixture reduced the potency of binding and inhibition. The compounds selected were as follows: tyrosol, hydroxytyrosol, oleuropein, ferulic acid, *p*-coumaric acid, vanillic acid, caffeic acid, apigenin, naringenin, and luteolin. Also included were cinnamic acid and syringic acid, which were not identified conclusively in the extracts here. However, previous work showed that EVOO contains cinnamic acid at a concentration (2–9 mg/kg) comparable to tyrosol and syringic acid at a concentration (<1 mg/kg) comparable to coumaric acid.^68^

**Figure 3:**
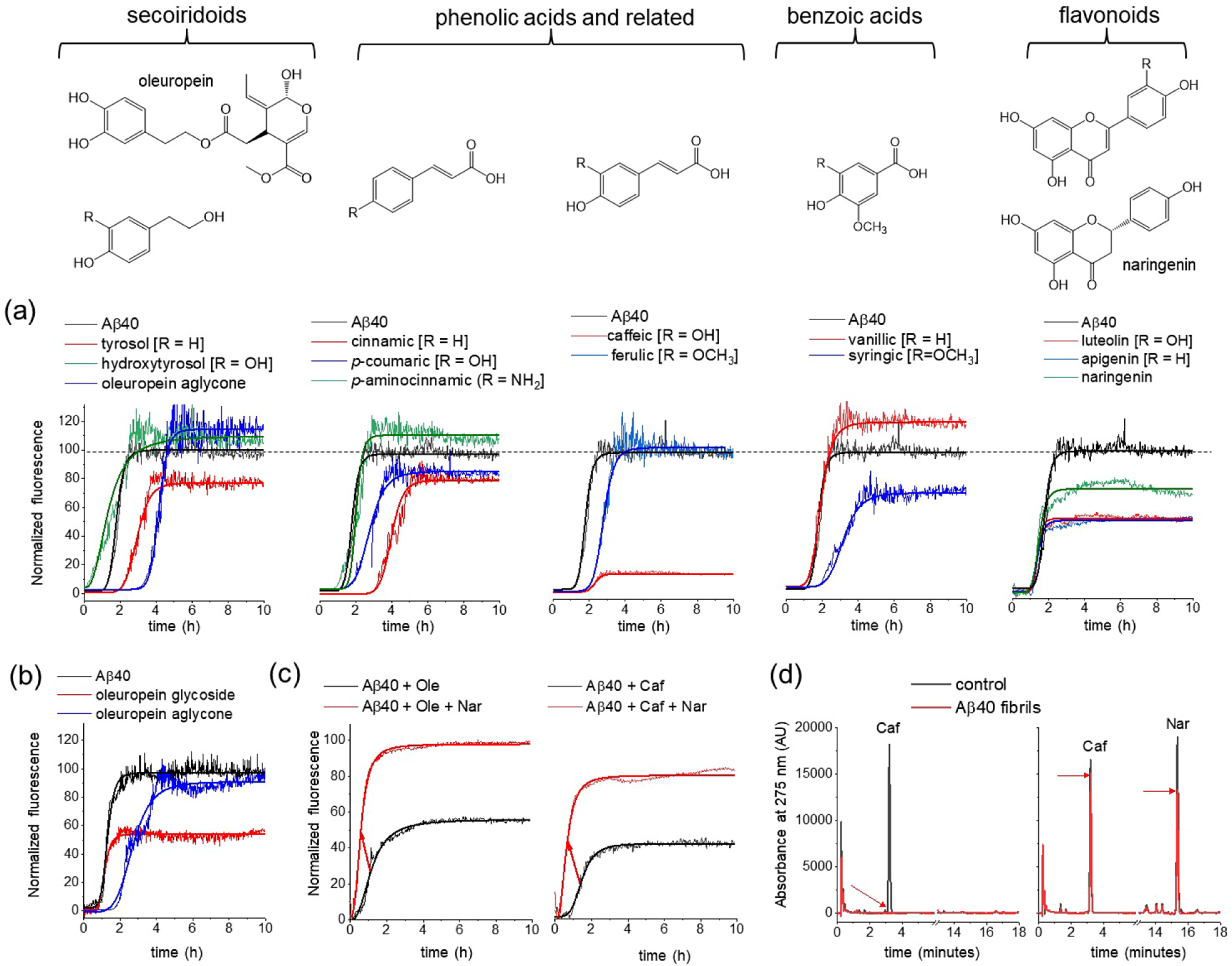
Effects on Aβ40 (20 μM) aggregation and fibril binding of standard EVOO phenolic compounds. (a) ThT analysis of Aβ40 aggregation kinetics, alone and in the presence of equimolar concentrations of individual polyphenols (chemical structures shown above). Means are shown of *n* = 3 measurements per group. (b) ThT analysis of Aβ40 aggregation, alone and in the presence of equimolar oleuropein aglycone and oleuropein glucoside. (c) ThT analysis of Aβ40 aggregation, in the presence of equimolar oleuropein aglycone (left) and caffeic acid (right) in the absence (black) and presence (red) of 20 μM naringenin. Red arrows indicate the increase in *t*_1/2_ in the presence of naringenin. (d) HPLC analysis of caffeic acid binding to Aβ40 fibrils in the absence and presence of naringenin (further details are given in the main text).

The effect of individual compounds on Aβ40 aggregation kinetics was monitored by thioflavin T (ThT) fluorescence over 10 h (Figure 3a and Table 3). ThT is an amyloid reactive dye, which displays enhanced fluorescence emission at ∼480 nm upon binding to amyloid structures. The individual compounds were added to monomeric Aβ40 in equimolar concentration (20 μM) and incubated with agitation at 37 °C during the fluorescence measurements. The ThT fluorescence curves exhibit a typical sigmoidal shape that reflects the increasing fibril concentration over time until the curve plateaus when aggregation is complete (Figure 3a).^69^ The data indicate that the individual compounds modify, to different extents, the maximum fluorescence, *F*_max_, at the completion of aggregation and *t*_1/2_, the time taken for fluorescence to reach half the value of *F*_max_ (Table 3). Although some compounds reduce *F*_max_, this does not necessarily indicate that the compounds reduce fibril yield. Some polyphenols have been shown to compete with ThT for fibrillar binding sites, and a reduced fluorescence can be falsely attributed to a reduction in fibril yield.^70^ In addition, some polyphenols may undergo spontaneous oxidation in an aqueous solution, generating compounds that strongly quench ThT fluorescence.^71^ For these reasons, the interpretation of *F*_max_ in terms of an inhibitory effect of the polyphenol extracts is unadvisible. However, the observed shifts in *t*_1/2_ are unlikely to arise from indirect effects of ThT and are more probably a result of direct interference of the extracts on Aβ40 aggregation. This conclusion is supported by previous work showing that the aggregation kinetics of Aβ40 are not affected by ThT at the concentration of 20 μM used here.^69^

**Table 3:** Effects of Selected EVOO Phenolic Compounds on Aβ40 and Tau Aggregation Kinetics (by ThT) and of Binding to Tau and Aβ40 Aggregates^a^^b^^c^

	ThT *F*_max_ (%)		ThT *t*_1/2_ (h)		% bound	
	Aβ40	tau	Aβ40	tau	Aβ40	tau
Aβ40 only	100.0 (9.2)		1.53 (0.23)			
tau only		100.0 (13.2)		2.23 (0.25)		
oleuropein	118.1 (12.3)	84.5 (12.1)	4.18 (0.26)	0.66 (0.14)	ND	ND
tyrosol	80.2 (8.1)	85.9 (9.2)	3.15 (0.22)	0.74 (0.31)	32.0	0.0
hydroxytyrosol	109.4 (11.3)	ND	1.32 (0.33)	ND	ND	ND
cinnamic acid	80.8 (12.9)	62.2 (11.3)	4.25 (0.21)	0.53 (0.27)	23.0	8.0
*p*-coumaric acid	86.6 (9.4)	100.3 (9.2)	2.96 (0.28)	2.03 (0.26)	12.0	0.0
ferulic acid	107.0 (9.9)	100.2 (14.3)	2.76 (0.29)	2.44 (0.34)	23.0	0.0
caffeic acid	12.8 (3.3)	47.7 (8.4)	2.34 (0.21)	1.90 (0.13)	49.0	16.0
vanillic acid	121.2 (11.0)	137.2 (0.0)	1.54 (0.22)	3.66 (0.13)	5.0	0.0
syringic acid	74.2 (9.3)	58.8 (19.2)	3.31 (0.34)	3.02 (0.34)	ND	ND
luteolin	50.5 (9.4)	ND	1.48 (0.17)	ND	41.0	ND
apigenin	49.2 (8.2)	ND	1.49 (0.13)	ND	38.0	ND
naringenin	71.3 (9.3)	ND	1.47 (0.1)	ND	4.0	ND

a The maximum fluorescence emission, *F*_max_, is expressed as a percentage of the value for Aβ40 or tau in the absence of extract.

b Means and standard errors (in parentheses) given for ThT data (*n* = 3).

c ND = not done.

Oleuropein aglycone and its metabolite tyrosol both shifted the *t*_1/2_ of the sigmoidal aggregation curves of Aβ40 to longer times, whereas hydroxytyrosol invoked a small decrease in *t*_1/2_ (Figure 3a). The phenolacrylic acids caffeic acid, *p*-coumaric acid, and ferulic acid also shifted the *t*_1/2_ of aggregation to longer times, as did cinnamic acid, which lacks the catechol hydroxyl groups. Interestingly, *p*-aminocinnamic acid, an amino analogue of *p*-coumaric acid that was tested as a model compound and was not identified in olive oil, had virtually no effect on *t*_1/2_ (1.48 h ± 0.32 h) compared to Aβ40 alone (1.53 h ± 0.23 h). The difference in the behaviors of *p*-aminocinnamic acid and *p*-coumaric acid implies that replacing the −OH group with -NH_2_ changes the size or hydrogen bonding capacity in such a way as to abolish the inhibitory effect. The hydroxybenzoic acid vanillic acid had no effect on *t*_1/2_, whereas the related syringic acid increased *t*_1/2_. The three flavonoids apigenin, luteolin, and naringenin had little effect on *t*_1/2_. These results indicate that EVOO phenols of different classes can have a very wide range of effects on Aβ40 aggregation kinetics.

The effect of oleuropein aglycone on *t*_1/2_ confirms previous reports that this compound reduces Aβ aggregation kinetics *in vitro*.^37^ However, EVOO contains various isomers and derivatives of oleuropein that have not been tested for amyloid inhibition, including its glucosyl derivative. Here, the ThT analysis of oleuropein glucoside indicates that it has little effect on *t*_1/2_ of Aβ40, in contrast to the inhibitory effect of oleuropein aglycone (Figure 3b). Interestingly, unlike the aglycone, the glucoside reduces *F*_max_, suggesting that it either reduces fibril yield or competes more effectively with ThT for fibril binding than does the aglycone. In EVOO, which contains a mixture of oleuropein aglycones and glucosides, the effectiveness of oleuropein in inhibiting aggregation may depend on the proportions of these compounds.

Next, it was investigated whether competition between different phenolic compounds for binding to Aβ40 could modify the effects on Aβ40 aggregation, as compared to the individual compounds. This possibility is relevant to EVOO phenolic mixtures, in which many compounds with different antiaggregation properties may compete for binding to Aβ40. ThT was used to monitor 20 μM Aβ40 aggregation in the presence of 20 μM caffeic acid or oleuropein aglycone, each in the absence or presence of 20 μM naringenin (Figure 3c). It was shown in Figure 3a that oleuropein aglycone and caffeic acid alone both increase *t*_1/2_, consistent with their reduction of aggregation kinetics, whereas naringenin alone does not affect *t*_1/2_. When naringenin is combined with oleuropein aglycone or caffeic acid, *t*_1/2_ is in both cases shifted to longer times, indicating that the presence of naringenin reverses the effects of the two inhibitory compounds. This reversal may be attributable to the noninhibitory naringenin competing with inhibitory oleuropein and caffeic acid for binding to Aβ40.

To confirm whether competition for binding to Aβ40 occurs, a reverse-phase HPLC method was developed to measure the binding of caffeic acid to Aβ40 fibrils in the absence and presence of naringenin. HPLC chromatograms were first obtained for a free caffeic acid (20 μM) solution and for caffeic acid combined with naringenin (20 μM each). Peaks for both compounds are fully resolved, with retention times of ∼3.4 min for caffeic acid and ∼15.6 min for naringenin (Figure 3d, black). The solutions were then added to Aβ40 fibrils (20 μM monomer equivalent) and centrifuged to remove the insoluble aggregates. The supernatants containing unbound caffeic acid and naringenin were analyzed by HPLC to reveal from the peak intensities how much of each compound had bound to the insoluble fibrils. For caffeic acid alone, the signature HPLC peak for caffeic acid had completely disappeared in the supernatant (Figure 3d, red), indicating that all the caffeic acid had bound to the fibrils and had been removed by centrifugation. By contrast, the supernatant peak for caffeic acid in the presence of naringenin reduced to ∼90% of the original intensity in the presence of naringenin. The peak for naringenin had also reduced to ∼70% of the initial intensity. The difference in the supernatant peak intensities for caffeic acid in the absence and presence of naringenin is consistent with competition between the two compounds for Aβ40 binding. Further work is underway to systematically determine the relative binding affinities of these and other phenolic compounds from the residual peak intensities.

To conclude this section, individual compounds identified in EVOO are shown to delay the aggregation of Aβ40. These compounds include caffeic acid, coumaric acid, tyrosol, and oleuropein aglycone. However, the alternative glucosyl form of oleuropein, also found in EVOO, does not delay Aβ40 aggregation. Furthermore, competition between inhibitory and noninhibitory phenolics for binding to Aβ40 reduces the inhibitory effects seen for individual compounds. The findings may have implications for the inhibitory capacity of the complex phenolic mixtures isolated from EVOO and justify why these mixtures should be tested alongside individual compounds.

### Effect of Individual EVOO Phenolics on Tau Aggregation Kinetics

**Figure 4:**
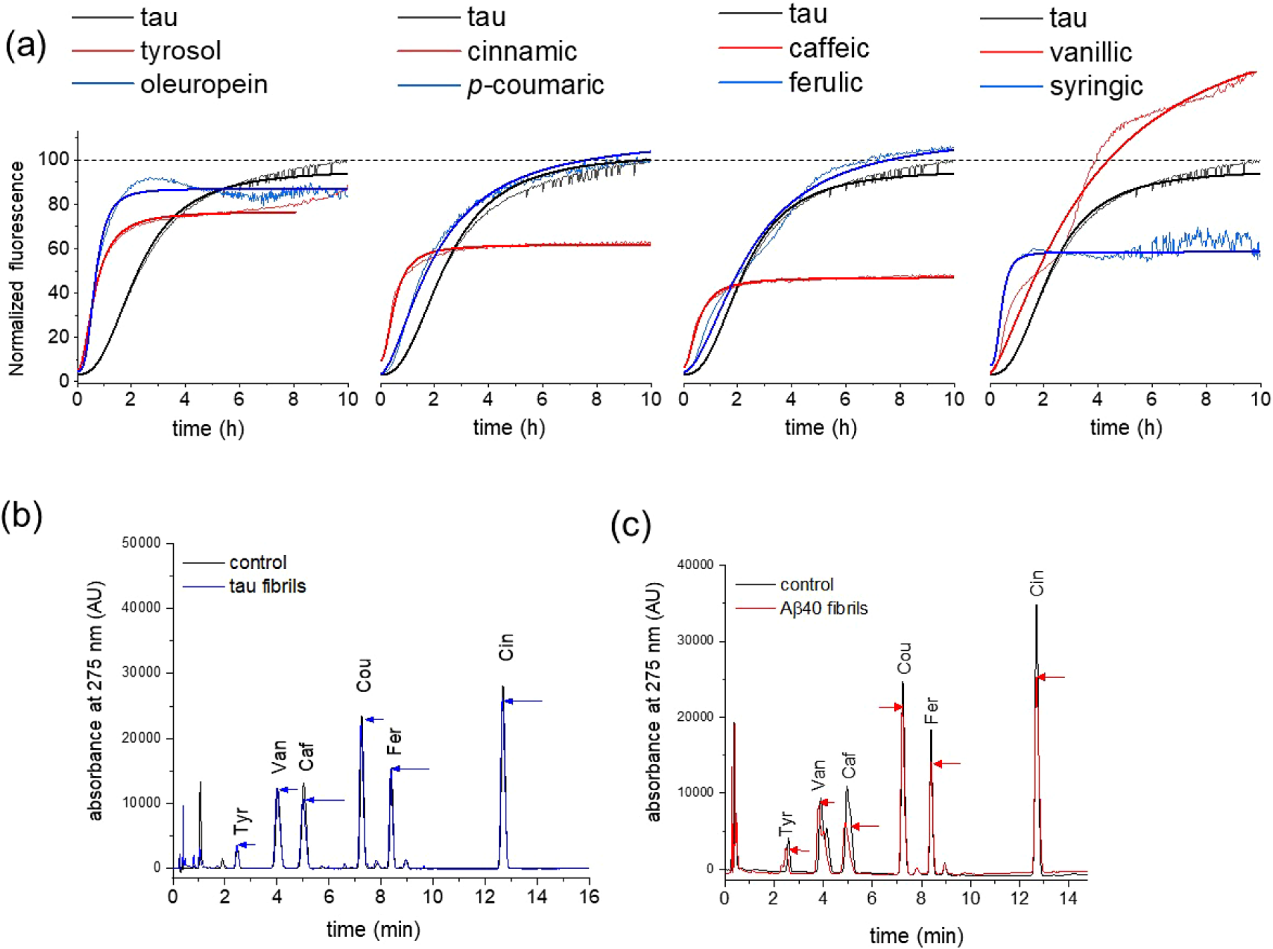
Effects on tau aggregation and fibril binding of individual EVOO phenolic compounds. (a) ThT analysis of tau (20 μM) aggregation kinetics, alone and in the presence of equimolar concentrations of individual polyphenols (chemical structures shown in Figure 3). Means are shown of *n* = 3 measurements per group. (b) HPLC binding analysis of a defined mixture of standard EVOO phenolic compounds in the presence of tau filaments. Chromatograms are shown for a solution of 20 μM compounds alone (black) and after addition of 20 μM tau followed by sedimentation (blue). (c) HPLC binding analysis of EVOO phenolic compounds in the presence of Aβ40 fibrils. Chromatograms are shown for two solutions of 20 μM standard compounds alone (black) and after addition and removal by the sedimentation of 20 μM Aβ40 (red). Arrows highlight the extent of peak reduction after sedimentation.

Several of the individual compounds tested against Aβ40 aggregation were also tested against a tau variant. The Δtau187 construct contains all the repeat domains, R1–R4, and in the presence of heparin is aggregation-prone without phosphorylation being necessary.^72^ Most studies of tau inhibition rely on nonphosphorylated constructs to avoid replicating the large and variable phosphorylation sites identified in tau *in vivo*. None of the compounds tested caused an appreciable reduction in the rate of tau aggregation but some compounds, including oleuropein, tyrosol, and caffeic acid, had the opposite effect and shortened *t*_1/2_ (Figure 4a and Table 3). It can be concluded, therefore, that at an equimolar concentration, the individual phenolic compounds are ineffective at reducing the rate of tau aggregation.

The HPLC method described in the previous section was used to assess the binding of a cocktail of the compounds (20 μM each) to tau fibrils (20 μM monomer equivalent). The compounds exhibited minimal binding to the insoluble filaments according to the reduction in HPLC peak intensities after removal of the fibrils (Figure 4b and Table 3). By contrast, when the cocktail was added to preformed Aβ40 fibrils (20 μM monomer equivalent), the peak intensities for the different compounds reduced to different extents after the addition and removal of Aβ40 fibrils, indicating that although the majority of the compounds bound to the fibrils, some (*e.g.*, caffeic acid) had higher affinity than others (Figure 4c and Table 3). The peak intensity reductions in the chromatogram likely reflect the competitive binding of the mixed compounds to the fibrils, as shown in the previous section. Hence, the selected EVOO phenolic compounds exert different inhibitory effects on tau and Aβ40 aggregation and bind to tau and Aβ40 fibrils to different extents.

### Effect of EVOO Phenolic Mixtures on Aβ40 and Tau Aggregation Kinetics

**Figure 5:**
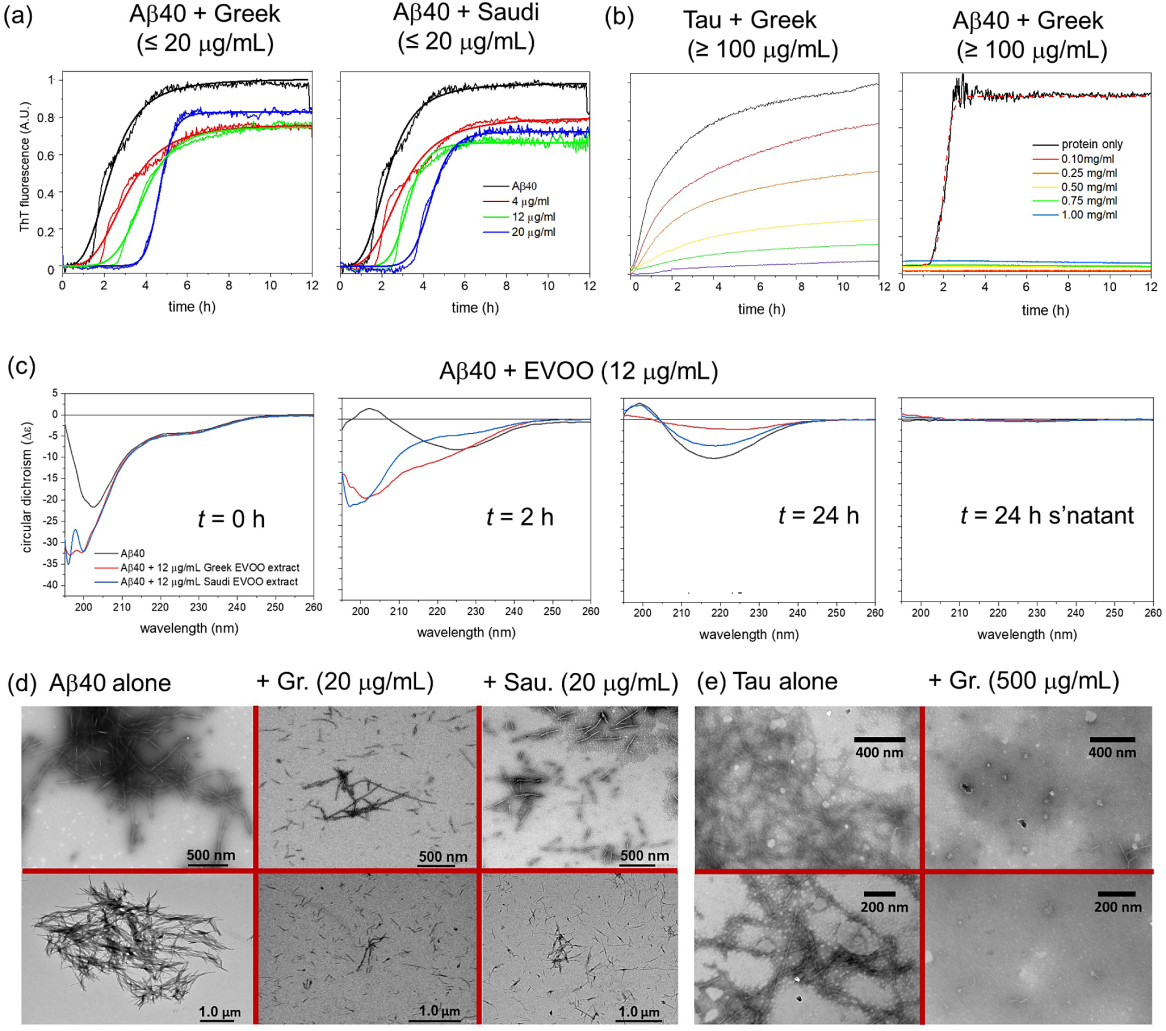
Kinetic and morphological analysis of tau and Aβ40 aggregation in the absence and presence of EVOO polyphenol extracts at different concentrations. The concentrations in mg/mL refer to the dry weight of the extracts dissolved in a solvent. (a) ThT analysis of Aβ40 aggregation in the absence and presence of low concentrations of the extract from Greek and Saudi EVOO. Lines of best fit using a standard Hill function with the values are shown in Table 3 . (b) ThT analysis of Tau and Aβ40 aggregation in the absence and presence of high concentrations (0.1–1.0 mg/mL) of the extract from Greek EVOO. Mean values of *n* = 3 measurements are shown. (c) Far-UV CD analysis of Aβ40 alone and in the presence of polyphenol extract from Greek and Saudi EVOO at *t* = 0, 2, and 24 h. The supernatant at 24 h was measured after sedimentation of insoluble material (right). (d) Negative-stain TEM images of Aβ40 aggregates (20 μM monomer equivalent) isolated after incubation alone or with 20 μg/mL (≅ 50 μM) Greek or 20 μg/mL Saudi EVOO extract for 3 days. (e) Negative-stain TEM images of tau aggregates (20 μM monomer equivalent) isolated after incubation alone or with 500 μg/mL (≅ 1.25 mM) Greek EVOO extract for 3 days. Two different magnifications and views are shown (top and bottom).

Next were examined the effects of the extracted EVOO phenolic mixtures on tau and Aβ40 aggregation kinetics. Aβ40 alone aggregates with a mean *t*_1/2_ of 2.3 h (Figure 5a, left, and Table 4). The Greek EVOO extract at concentrations up to 20 μg/mL (equivalent to an average molar concentration of ∼50 μM) had a progressive effect on the aggregation rate of Aβ40 (Figure 5a, left). Increasing the extract concentration from 4 to 20 μg/mL shifted *t*_1/2_ from 2.3 to 4.7 h (Table 4). The Saudi extract had a very similar effect at these concentrations, despite having a different phenolic profile to the Greek extract (Figure 5a, right). The maximum fluorescence, *F*_max_, observed at the end-point was in all cases significantly lower in the presence of the extracts than for Aβ40 alone, but there was no significant difference between *F*_max_ values for Aβ40 in the presence of extracts at different concentrations. Against tau, much higher concentrations of EVOO mixture (≥100 μg/mL; ∼ ≥250 μM) were needed to observe an effect on aggregation (Figure 5b, left). At these high concentrations, the Greek EVOO extract caused a progressive reduction in ThT fluorescence at the measurement end-point of 12 h. A closer inspection of the data reveals that increasing the EVOO extract concentrations lengthens the lag time and decreases the rate of filament elongation (Figure S3). Typical studies of amyloid inhibition by pure compounds focus on subequimolar concentrations with respect to the protein concentration (20 μM in this case).^28^ Therefore, although the phenolic mixture in the extract is capable of reducing the tau aggregation rate, it requires a 10-fold higher concentration than is generally regarded as suitable for an inhibitory compound. For comparison, Aβ40 aggregation is virtually abolished in the presence of the Greek EVOO extract at the same concentrations used for tau inhibition (Figure 5b, right). The results mirror the effects of individual EVOO compounds in that Aβ40 inhibition is much more effective at lower phenolic concentrations than is tau aggregation.

**Table 4:** Summary of the Effect of EVOO Extracts on Aβ40 Aggregation Kinetics as Assessed by ThT Fluorescence^a^^b^

extract	extract concentration							
	Aβ40 only		4 μg/mL		12 μg/mL		20 μg/mL	
	*F*_max_	*t*_1/2_ (h)	*F*_max_	*t*_1/2_ (h)	*F*_max_	*t*_1/2_ (h)	*F*_max_	*t*_1/2_ (h)
Greek	1.00 (0.15)	2.31 (0.20)	0.76 (0.18)	3.06 (0.22)	0.75 (0.13)	3.90 (0.32)	0.83 (0.15)	4.67 (0.25)
Saudi	1.00 (0.15)	2.31 (0.20)	0.80 (0.19)	2.90 (0.25)	0.67 (0.19)	3.25 (0.23)	0.73 (0.16)	4.37 (0.13)

a The maximum fluorescence emission, *F*_max_, is expressed normalized to Aβ40 in the absence of extract.

b Means (standard errors) are given from measurements on *n* = 3 samples per group.

Further techniques were used to confirm that the extracts do indeed impede Aβ40 aggregation. Far-UV circular dichroism spectroscopy was used to monitor the transitions of the Aβ40 secondary structure during aggregation (Figure 5c). The initial spectrum of Aβ40 at *t* = 0 has the features expected for an unfolded protein (*e.g.*, a large negative lobe at ∼200 nm). Interestingly, a larger negative Δε is observed at 200 nm in the presence of the Greek and Saudi extracts (12 μg/mL) than for Aβ40 alone, even though the background spectra of the extracts had been subtracted. This observation suggests that Aβ40 alone may undergo partial folding in the short period between sample preparation and recording the first spectrum and that the EVOO extracts stabilize the initial unfolded state during this period. The spectra of Aβ40 alone at *t* = 2 h are consistent with a partial transition to a β-sheet-containing state (*i.e.*, a positive lobe at 200 nm and a negative lobe at ∼222 nm). In the presence of the Greek and Saudi EVOO extracts, the spectra retain a negative lobe around 200 nm suggesting that a proportion of Aβ40 retains the initial unfolded state. After 24 h incubation, the spectra all exhibit a negative lobe at ∼220 nm and lose the negative lobe at ∼200 nm, which is consistent with complete loss of the initial state and the formation of β-sheet structures in all cases. Although both EVOO extracts evidently interfere with Aβ40 aggregation, there are differences in the spectra at *t* = 2 h and *t* = 24 h that suggest that the Saudi and Greek extracts have different effects on Aβ40 aggregation kinetics and/or structural content. For instance, the variability in Δε at 222 nm after 24 h may arise from different spectral proportions of left-handed and right-handed β-sheets, which cancel each other to different extents. There is little or no signal from the supernatant after removal of insoluble aggregates by centrifugation at the end-point, suggesting that aggregation in all cases had reached completion (Figure 5c, right).

Negative-stain transmission electron microscopy (TEM) was used to visualize the morphology and extent of deposition of tau and Aβ40 aggregates formed in the presence of the EVOO extract mixtures (Figure 5d,e). A fresh solution of monomeric Aβ40 (20 μM) was incubated alone or with the Greek or Saudi extracts (20 μg mL ≅ 50 μM) for 24 h. Aggregation of Aβ40 alone resulted in fibrillar species with a width of 17.7 (±0.4) nm and a length of 396 (±25) nm, clustered together in dense networks (Figure 5d, left). In the presence of the EVOO extracts from Greek and Saudi sources, the resultant fibrils are seen to be distributed much more sparsely and are shorter and more slender than those formed in the absence of the extract (Figure 5d, middle and right). No significant populations of nonfibrillar structures can be observed, and there is no discernible difference between the extent of fibril deposition or morphology in the presence of the two extracts. Incubation of tau over the same period resulted in fibrillar structures (Figure 5e) that were not distinguishable from fibrils obtained with 20 μg/mL EVOO in terms of their density and morphology (data not presented). However, the tau morphology was altered when incubated with 0.5 mg/mL EVOO extract, which was shown to be sufficient to elicit a reduced ThT fluorescence in the presence of tau (Figure 5b). This higher extract concentration resulted in the formation of spherical oligomers along with filaments similar in size and morphology to those of tau alone.

To summarize, both the Greek and Saudi EVOO extracts reduce the rate of Aβ40 aggregation as assessed by ThT and CD spectroscopy, in the concentration range around that of AβBy contrast, at least a 10-fold higher concentration of the extracts is required to inhibit tau aggregation. The Greek and Saudi extracts have similar effects on Aβ40, despite the Saudi extract having a lower concentration of oleuropein aglycone, tyrosol, and hydroxytyrosol than the Greek extract. This suggests that other phenolic compounds in the Saudi extract may compensate for the deficiency in these known inhibitory compounds.

### Effect of EVOO Phenolic Extracts on Preformed Aβ40 and Tau Aggregates

It was next investigated whether the phenolic mixtures can remodel preformed Aβ40 and tau fibrillar aggregates into alternative morphologies. Amyloid-remodeling behavior has been observed for certain individual phenolic compounds, most notably the green tea polyphenol, EGCG.^73,74^ Proteins (Aβ40 and tau at 20 μM) were each incubated alone for 3 days, after which time the insoluble aggregates were isolated by centrifugation. EVOO extract solutions were added to the sedimented aggregates to a final concentration of 20 (≅ 50 μM), 72, or 740 μg mL and incubated for a further 24 h before separating the insoluble and soluble fractions by centrifugation.

**Figure 6:**
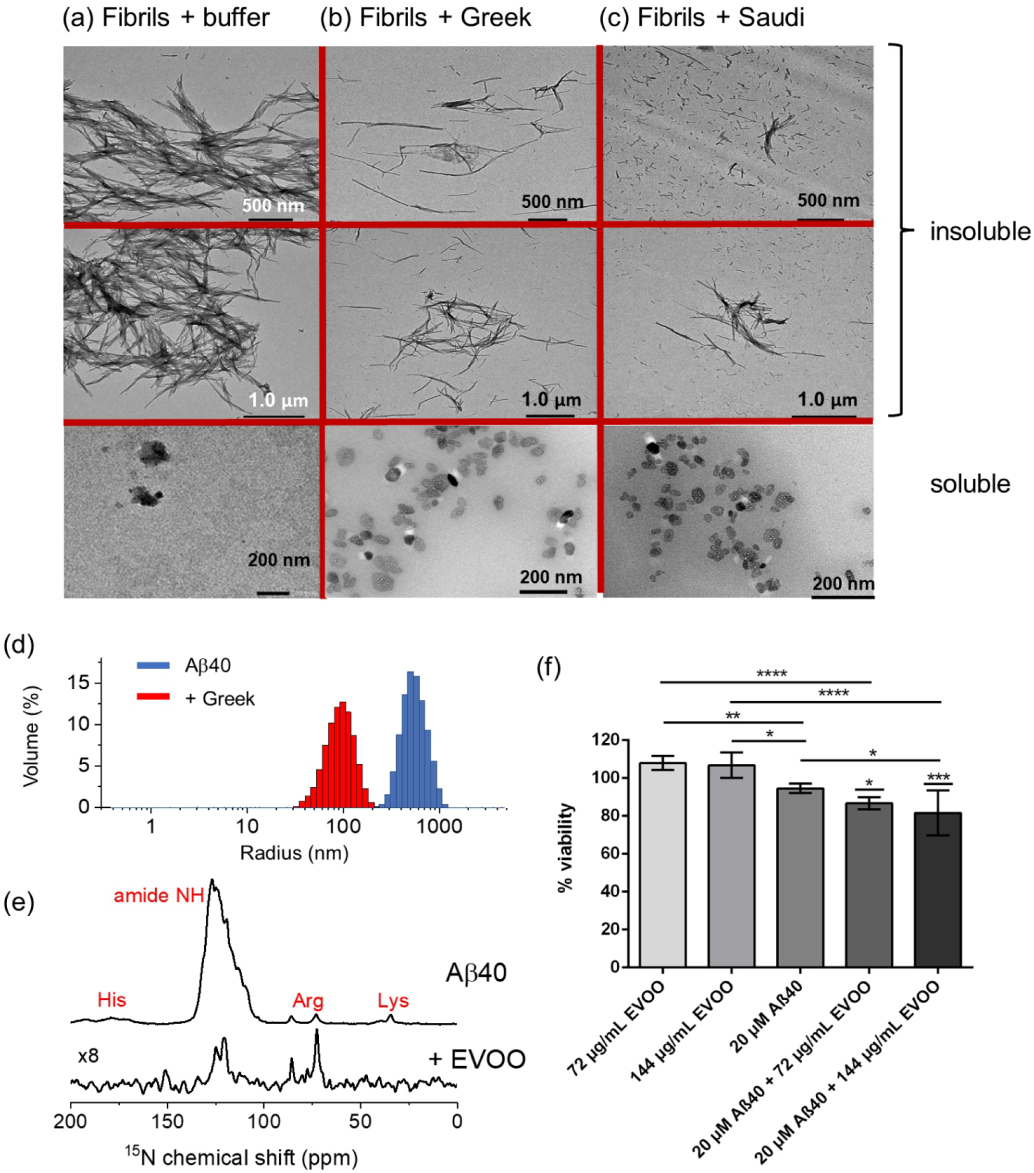
Negative-stain TEM images of preformed Aβ40 fibrils (20 μM monomer equivalent) incubated for 24 h with either buffer or with 20 μg/mL (≅ 50 μM) EVOO extract. The TEM images show the soluble and insoluble fractions obtained after centrifugation. (a) Aβ40 incubated with buffer. (b) Aβ40 in the presence of Greek EVOO extract. (c) Aβ40 in the presence of Saudi EVOO extract. Two different magnifications and views are shown. (d) DLS data for Aβ40 fibrils alone and following the addition of 740 μg/mL Greek EVOO. (e) ^15^N CP-MAS (top) and refocused ^1^H–^15^N INEPT spectra of [U–^15^N]Aβ40 fibrils treated with Greek extract. (f) Viability data for SH-SY5Y cells following the addition of EVOO, Aβ40 fibrils alone, and following the addition of 72 μg/mL and 144 μg/mL Greek EVOO, *n* = 6 per condition. *p*-values were determined using ANOVA with Tukey’s multiple comparison correction between live and Aβ40-treated cells in the presence of EVOO at both concentrations and between relevant comparison groups as shown. *p < 0.05, **p < 0.01, ****p < 0.001, ****p < 0.0001.

Aβ40 alone deposited a dense network of fibrils (Figure 6a top and Figure 5d, left), and virtually no soluble material was observed in the supernatant after centrifugation (Figure 6a, bottom). Preformed fibrils treated with 20 μg/mL EVOO extract solutions (Greek and Saudi) and sedimented by centrifugation are seen to remodel into slender insoluble fibrils (Figure 6b,c). In the supernatant of the same sample, minor populations of soluble annular or spherical structures averaging ∼30 nm in diameter and reminiscent of oligomers^75^ can be seen. These species constitute a very small fraction of the total aggregate mass at this extract concentration and were completely absent when extracts were added at concentrations <20 μg/mL (data not shown). The addition of 72 μg mL of Greek extract solution increased the number of soluble oligomers, and after the addition of 740 μg/mL of Greek extract, virtually all the detectable aggregates had remodeled into the oligomeric morphology, albeit with a smaller average diameter (Figure S4). Tau forms dense filaments after incubation for 3 days in the absence of the extracts. The addition of the Greek extract solution at concentrations up to 500 μg/mL to the preformed tau filaments had virtually no effect on the morphology of the aggregates (Figure S5).

Further measurements were conducted on the preformed Aβ40 aggregates after the addition of the Greek extract. Dynamic light scattering (DLS) indicated that the addition of the highest extract concentration (740 μg/mL) reduced the mean diameter of the aggregates by almost an order of magnitude (Figure 6d). ^15^N cross-polarization magic-angle spinning solid-state NMR of uniformly ^15^N-labeled Aβ40 fibrils before treatment with the extract exhibits characteristic peaks from the backbone amide (100–125 ppm) and arginine, lysine, and histidine side-chains (Figure 6e, top). The CP-MAS spectrum displays peaks only from dynamically restricted sites and is consistent with intact fibrils. A ^1^H–^15^N refocused INEPT SSNMR experiment on the same sample (not shown) did not detect any signals, but after the addition of the EVOO extract (20 μg/mL), selective peaks from the backbone and arginine ^15^N sites emerged in the INEPT spectrum (Figure 6e, bottom). The INEPT experiment detects resonances only from mobile groups and is consistent with a partial mobilization of the fibrils after the addition of the extract. Together, the TEM, DLS, and SSNMR data indicate that the phenolic extracts mobilize Aβ40 fibrils to form soluble oligomers while remodeling the remaining fibrils to form more slender structures.

Soluble oligomers of Aβ40 that form on-pathway to the mature fibrils have been extensively reported as being associated with cellular toxicity.^13^ The cytotoxicity of the oligomer-like species formed by Aβ40 in the presence of the Greek phenolic extract was assessed in a cell viability assay with SH-SY5Y neuroblastoma cells. For this experiment, the effects of the extract at concentrations of 72 μg/mL and 144 μg/mL were assessed. These concentrations are high enough to promote the formation of oligomers and substantially higher than required to completely abolish aggregation but not so high as to completely solubilize the fibrils. The extract solutions alone had no effect on cell viability at the two concentrations (Figure 6e). In the absence of the extract solution, Aβ40 aggregates formed after 3 days (total soluble and insoluble fractions) had a small (<10%) reduction in cell viability. Treatment of the Aβ40 aggregates with the extract solutions for 24 h before addition to the cells reduced the cell viability by a further 5–10% compared to Aβ40 alone. It can therefore be concluded that the addition of extracts at these concentrations to Aβ40 aggregates promotes the further formation of cytotoxic species, consistent with the remodeling into oligomers observed by TEM.

### Binding of EVOO Extracts to Aβ40 Fibrils and Tau Filaments

The ability of the EVOO extracts to remodel Aβ40 fibrils into slender, shorter structures and soluble oligomers indicates that some components of the extract mixtures must interact with the aggregates. We therefore investigated which of the phenolic compounds in the EVOO cocktail bind to the insoluble Aβ40 species, using UV–visible spectroscopy to estimate the binding of the entire mixture and the HPLC method to resolve individual species. The extracts (20 μg/mL) and preformed fibrils (20 μM monomer equivalent) were incubated for 24 h, and then, the insoluble material was removed by sedimentation. The concentration of phenolic compounds remaining in the supernatant was determined to find differences in the concentration of compounds that bind to the insoluble fibrils and cosediments with them. It should be recalled that, at this concentration, the extracts generate a small population of soluble oligomers, so the EVOO compounds remaining in the supernatant may not be “free” but bound to the small population of soluble amyloid species.

**Figure 7:**
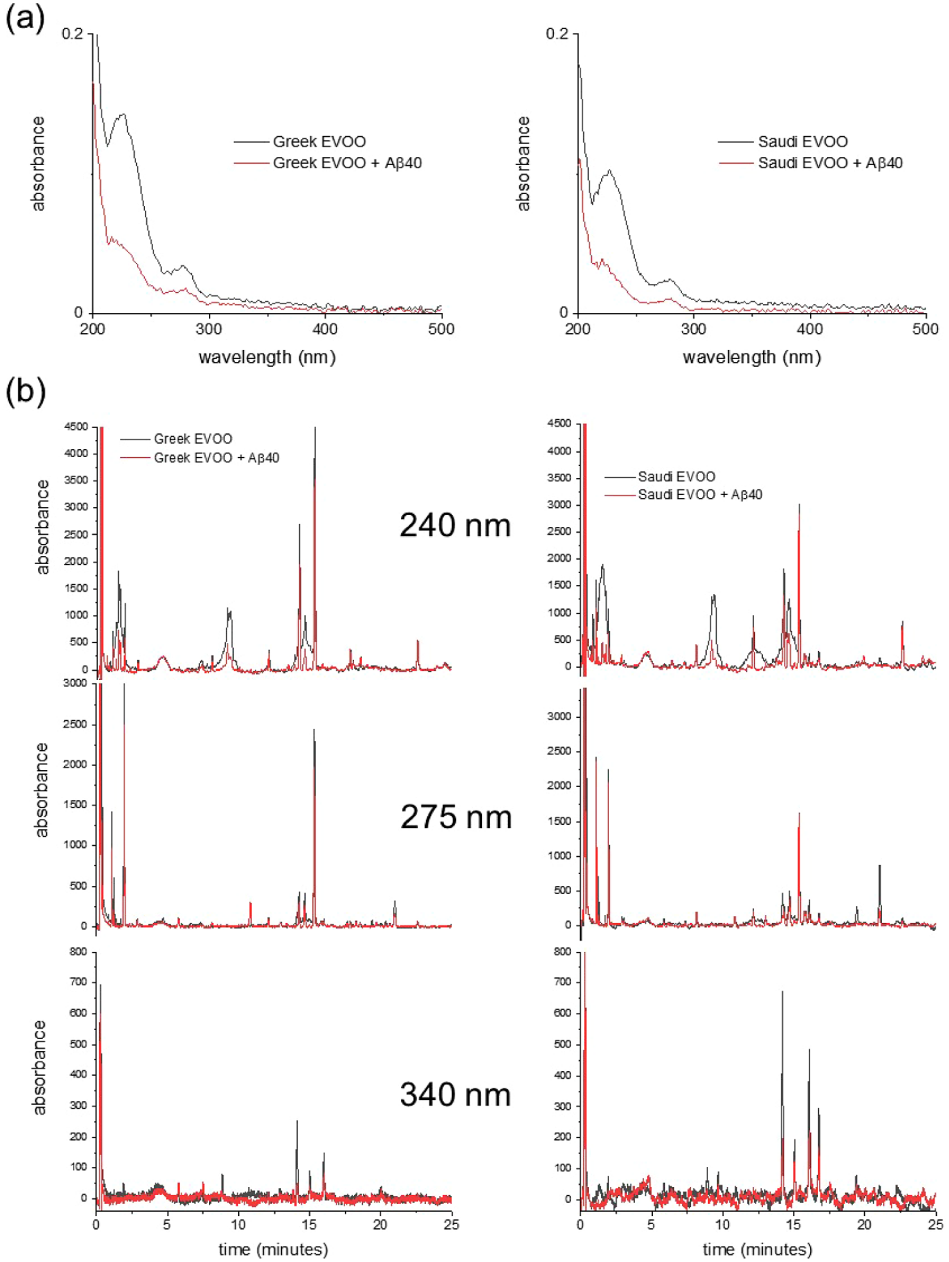
Binding of phenolic compounds in the EVOO extracts to preformed fibrils of Aβ40. (a) UV–visible absorption spectra of Greek (left) and Saudi (right) extracts (20 μg/mL) alone (black lines) and of the supernatant obtained after the addition of 20 μM Aβ40 fibrils, incubation for 24 h, and removal of insoluble material by centrifugation (red lines). (b) Reverse-phase HPLC chromatograms at 3 nm of the Greek (left) and Saudi (right) extracts alone (black) and after the addition and removal of Aβ40 fibrils (red). The main peaks, retention times, and some assignments are given in Table 5.

Figure 7a shows the UV–visible spectra of the Greek and Saudi extract solutions alone and after the addition of Aβ40 fibrils and subsequent removal by sedimentation. The absorption across the entirety of the spectra is seen to reduce by >50% after the addition and removal of the fibrils, indicating that a large proportion of the species in the extracts bind to the insoluble fibril fraction. The binding species in the Greek and Saudi extracts were resolved by reverse-phase HPLC (Figure 7b, Tables 5 and S2), which indicated that the vast majority of the compounds detectable at 240, 275, and 340 nm bound to the fibrils to some extent and, in some cases, were removed from solution completely. Peaks that were reduced in intensity included the broad peaks attributed in Figure 1b to elenolic acid, oleuropein isomers and derivatives, pinoresinol and ligstrosides, and sharper peaks more prominent at the longer wavelengths, including from tyrosol and flavonoids. All the peaks assigned to the reference standards were reduced in intensity (Table 5, shaded rows), as were many more unassigned peaks. As discussed earlier, competitive binding of the individual phenols in the mixture will influence the extent to which the peaks are reduced. Nevertheless, the conclusion is that many, if not all, of the phenolic compounds present in the Greek and Saudi extracts bind to some extent to the Aβ40 fibrils.

**Table 5:** Summary of the HPLC Analysis of EVOO Compound Binding (20 μg/mL Greek Extract) to Insoluble Aβ40 Fibrils (20 μM Monomer Equivalent)

retention time (min)	compound	normalized peak intensity^a^		% bound	λ (nm)^b^
		–fibril	+fibril		
1.0	hydroxytyrosol	12.9	3.8	71	275
1.2	unknown	11.7	1.7	86	275
1.7	unknown	1.3	0.0	100	275
1.9	tyrosol	93.1	8.4	91	275
2.9	vanillic acid	2.2	0.0	100	275
3.0	caffeic acid	1.6	0.0	100	275
5.7	*p*-coumaric acid	3.8	0.0	100	275
8.1	unknown	7.2	6.3	12	240
8.9	unknown	1.4	0.0	100	340
9.2	unknown	100.0	14.4	86	240
9.4	unknown	96.5	6.3	93	240
12.1	unknown	6.5	5.5	16	240
14.1	luteolin	3.6	1.2	66	340
14.3	unknown	11.8	7.2	39	275
14.6	(+)-pinoresinol	12.9	8.9	31	275
15.0	unknown	2.1	0.0	100	340
15.3	naringenin	70.3	55.5	21	275
15.8	unknown	2.4	0.0	100	275
16.0	apigenin	3.1	0.0	100	340
17.7	unknown	1.5	0.0	100	275
17.8	unknown	1.6	1.2	24	275
18.3	unknown	2.5	2.0	25	275
19.7	unknown	3.2	0.0	100	275
19.9	unknown	1.1	0.0	100	275
20.4	unknown	1.4	0.0	100	275
22.6	unknown	20.2	14.6	28	240

a Normalized to the maximum peak intensity (at 9.2 min) in the absence of fibrils.

b Wavelength of maximum absorbance chosen for quantification.

**Figure 8:**
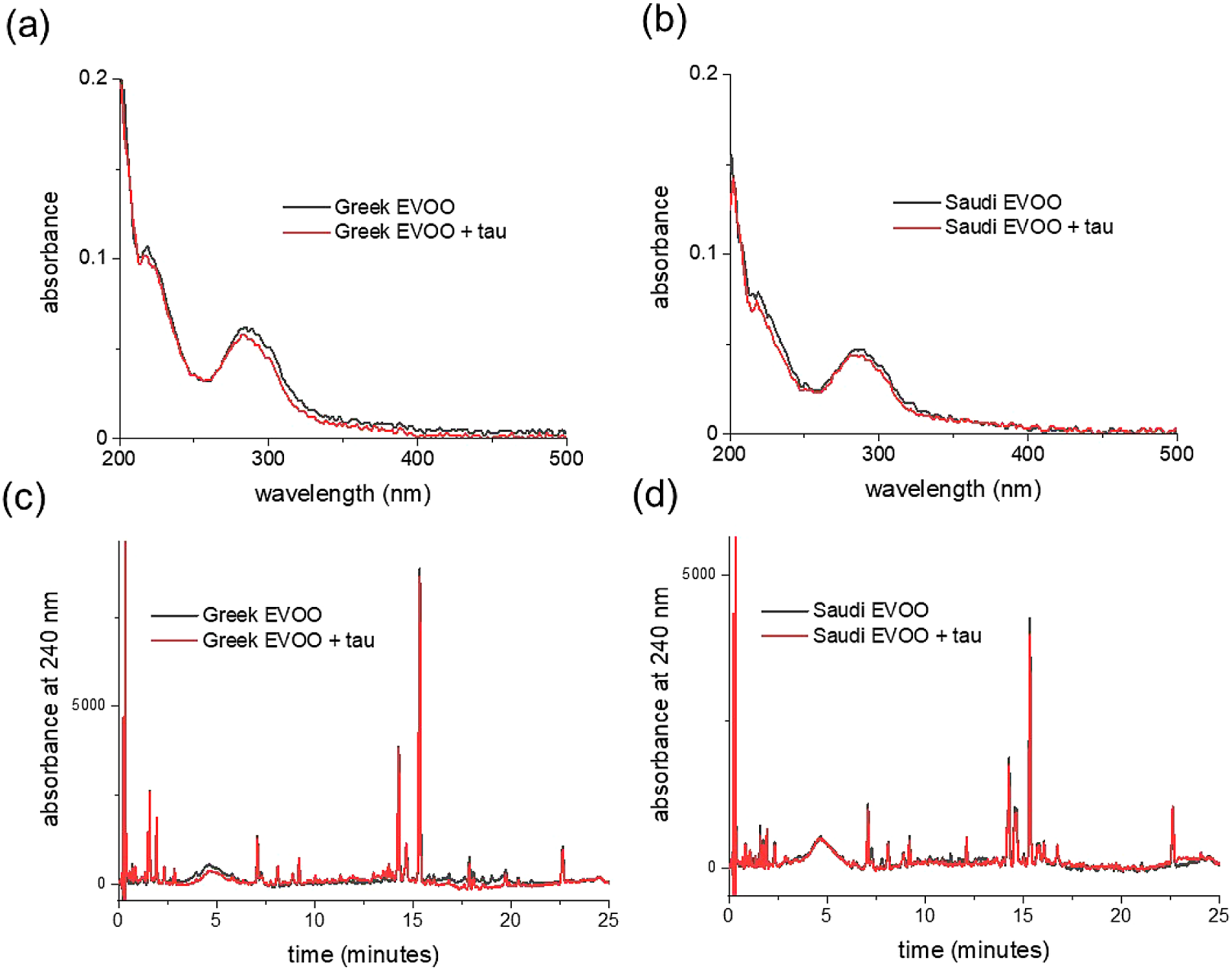
Binding of phenolic compounds in the EVOO extracts to preformed fibrils of tau. (a, b) UV–visible absorption spectra of Greek and Saudi extracts (20 μg/mL) alone (black lines) and of the supernatant obtained after the addition of 20 μM tau fibrils, incubation for 24 h, and removal of insoluble material by centrifugation (red lines). (c, d) Reverse-phase HPLC chromatograms at 240 nm of the Greek and Saudi extracts alone (black) and after the addition and removal of tau fibrils (red).

In contrast to the extensive binding of the polyphenol mixtures to Aβ40 aggregates, the UV–vis and HPLC analysis of the extracts in the presence of tau aggregates showed little or no binding (Figure 8). This negative result is consistent with the lack of effect of the extract solution on tau filament morphology and on the reduced efficiency at inhibiting tau aggregation. Interestingly, the contrast between the binding to the Aβ40 and tau aggregates argues against nonspecific binding and suggests that specific recognition sites for polyphenols are present on the Aβ40 fibrils that are absent from the tau filaments. It should, however, be noted that tau requires the presence of polyanionic species to aggregate (in this case heparin), which may influence the binding.

## Discussion and Conclusions

### EVOO Polyphenol Mixtures Inhibit Aβ40 Aggregation

The Mediterranean diet has been widely promoted as being protective against several pathological diseases, including AD,^1−4^ which affects 44 million people worldwide and has a predicted economic burden of $600 billion in the US alone by 2050.^76^ Several preclinical and epidemiological studies have linked the consumption of EVOO in particular to the amelioration of AD symptoms.^77^ Positive effects of an EVOO-enriched diet were demonstrated in a triple transgenic (3xTg) AD mouse model expressing both Aβ and tau pathologies, including enhanced behavioral performance, reduced insoluble Aβ deposition, and decreased tau phosphorylation.^78^ Clinical studies of EVOO intervention in patients with mild cognitive impairment (MCI), an early indicator of AD, have shown potential benefits. EVOO restores levels of the neuroprotective protein BMI1 in MCI patients, constituting a potential therapeutic approach against neurodegeneration leading to AD.^79^ Long-term intervention with EVOO was also associated with significant improvement in cognitive function in patients with MCI.^80^ Further, EVOO consumption in MCI patients attenuates oxidative and nitrative stress reflecting on the reduction in the PARP levels and DNA damage.^81^ Moreover, EVOO significantly reduced blood Aβ42/Aβ40 and phosphorylated-tau/total-tau ratios in a small cohort of MCI patients, suggesting that olive oil alters the processing and clearance of Aβ.^82^

Individual polyphenols identified in EVOO, particularly oleuropein and (hydroxy)tyrosol, have attracted attention because of their ability to disrupt the formation of Aβ and tau amyloid species that present in the AD brain as plaques and neurofibrillary tangles, respectively.^14,19^ Until now, however, it has not been investigated whether the complex phenolic mixtures that occur in dietary EVOO can also, collectively, interfere with Aβ and tau aggregation. As set out in the Introduction, there are several reasons why phenolic mixtures in EVOO may differ from individual components in their ability to modify Aβ40 aggregation. Without experimental confirmation, it cannot be taken for granted that EVOO phenol mixtures have the same effects on Aβ peptides as do individually active compounds.

In this work, we characterized phenolic extracts prepared from two EVOO sources and confirmed that they have distinct compositions, containing different concentration profiles of oleuropein and its metabolites, phenolic acids, and flavonoids. In both samples, ^1^H NMR spectroscopy indicated the presence of different phenolic isomers and glucosyl derivatives. Certain compounds identified in the mixtures, including oleuropein aglycone, were shown to individually reduce Aβ40 aggregation rates when in equimolar concentration with the peptide. However, the glucosyl derivative of oleuropein did not affect Aβ40 aggregation kinetics, suggesting that the balance of oleuropein isomers and derivatives in EVOO might be important for reducing amyloid aggregation. Further testing of mixtures of the compounds caffeic acid, oleuropein aglycone, and naringenin confirmed that competitive binding can reverse the effect of compounds that are alone inhibitory.

Phenolic mixtures extracted from EVOO contained several different isomers and derivatives of oleuropein and its metabolites, as well as nonactive compounds such as flavonoids that could compete with inhibitory compounds for binding to Aβ40. Nevertheless, the EVOO phenol mixtures were found to reduce the rate of Aβ40 aggregation by lengthening *t*_1/2_ in a concentration-dependent manner. The overall concentration of phenols in the mixture (up to 20 μg/mL) that produced this effect was similar to the active concentration window of the individual compounds, including oleuropein aglycone. Interestingly, the Greek and Saudi extracts were shown to be similarly effective at inhibiting Aβ40 aggregation *in vitro*, despite having different phenolic profiles. This finding can be rationalized by attributing the inhibitory efficacies of different EVOO mixtures to a collective effect of the entire phenolic pool rather than to the concentrations of individual compounds that are known to be active, such as oleuropein aglycone, tyrosol, and hydroxytyrosol. Hence, deficiencies in certain phenolics in a sample may be buffered by higher, compensatory concentrations of others such that extracts of different compositions can have similar antiaggregation properties. This argument is supported by the HPLC binding analysis, which reveals that the vast majority of EVOO compounds bind to Aβ40 fibrils.

The ability of the EVOO mixtures to interact with Aβ40 fibrils and remodel them into soluble oligomers is a property of other polyphenols from food sources, including the flavonoid kaempferol, EGCG from green tea, and resveratrol from grapes.^83−85^ Unlike the amyloid oligomers promoted by these latter compounds, we found that the EVOO phenol-induced Aβ40 oligomers are mildly cytotoxic to SH-SY5Y cells; EGCG and resveratrol remodel fibrils and oligomers into nontoxic, off-pathway species. Although relatively low concentrations of the extracts (20 μg/mL) are required to generate Aβ40 oligomers, the oligomers represent minor populations of the overall amyloid species. However, pathological consequences could arise if cytotoxic amyloid oligomers accumulated *in vivo* as a result of regular EVOO consumption. This possibility is counter to the health benefits of EVOO but warrants further investigation.

### Why Do EVOO Polyphenols Interact Weakly with Tau?

It was surprising that the EVOO extract mixtures were considerably less efficient at reducing tau aggregation than they were at reducing Aβ40 aggregation. Much higher concentrations were required to observe an effect on tau aggregation kinetics than were normally considered suitable for an inhibitory compound. The poor inhibitory effect on tau paralleled the weaker overall binding of the EVOO phenolics to tau filaments. Selected EVOO compounds also had little or no inhibitory effect on tau and possibly promoted aggregation. Furthermore, much higher concentrations (500 μg/mL) were required to remodel the tau filaments into oligomers than were needed to generate Aβ40 oligomers. The clear difference in tau and Aβ40 inhibition and binding argues against nonspecific interactions of the phenolic mixture with the proteins in their various stages of aggregation and suggests instead that specific binding sites exist in the Aβ40 aggregates that are absent from tau.

The reasons for the different effectiveness of the EVOO mixtures against Aβ40 and tau can only be speculated upon without experimental mechanistic studies, which are beyond the scope of the present work. One possibility is that heparin, which is a polyanionic molecule needed to induce tau aggregation *in vitro*, is incorporated into the tau fibrils and repels interactions with phenolic molecules. However, heparin is also required to accelerate the amyloid formation of apoA-I but does not prevent interactions of the fibrils with the polyphenol EGCG.^73^ The answer may therefore lie in the different fibrillar architectures of Aβ40 and tau. Many of the compounds extracted from EVOO have a preference for β-sheet structures, as confirmed by inspecting the structures of protein–phenol complexes from the Protein Data Bank (PDB). The phenolic compounds ferulic acid, apigenin, coumaric acid, caffeic acid, luteolin, and others bind predominantly to β-sheet regions, even in proteins having a high α-helical content (Figure S6a,b).

Polyphenols may bind to proteins through reversible noncovalent interactions or nonreversibly through covalent bonding (reviewed in the previous study).^86^ It has been suggested that the inhibition of amyloid formation may involve noncovalent stabilizing interactions between phenolic and protein aromatic groups, possibly enabled by the ability of planar aromatic groups to insert between, or align with, β-sheet layers.^28,87^ The phenolic rings of polyphenol compounds interfere with π-stacking, thus inhibiting the stabilization of the amyloid core structure,^36^ with the hydroxyl groups contributing to disruption of the hydrophobic core and increasing solubility.^37,38^ However, other mechanisms can drive phenol–protein interactions in general, including hydrogen-bonding, hydrophobic interactions, and van der Waals interactions.^86^

**Figure 9:**
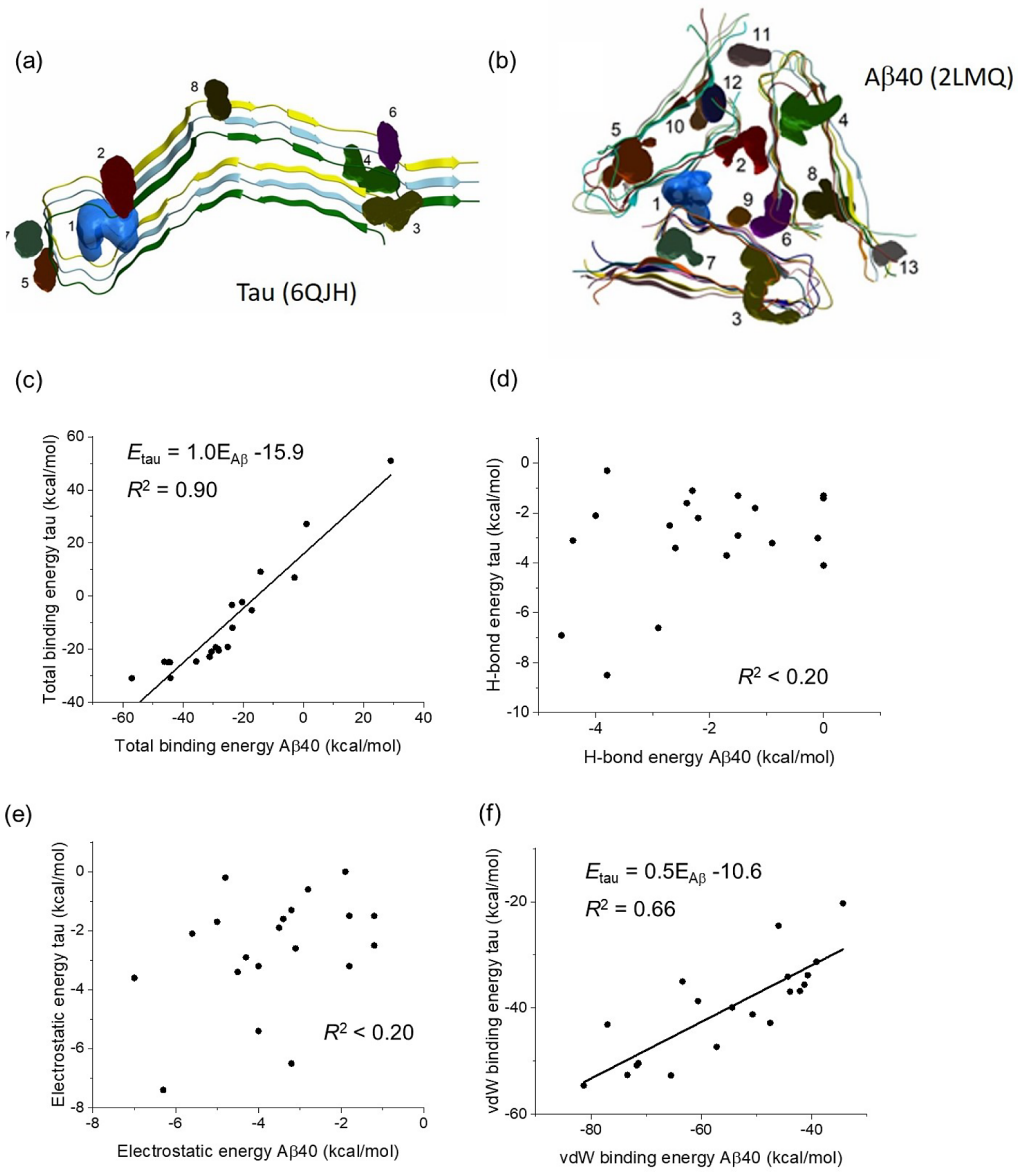
Computational docking analysis of EVOO phenolic compounds with Aβ40 and tau fibrils. (a) Structural model of heparin-induced tau filament core from cryo-electron microscopy,^88^ showing drug binding pockets 1–8 predicted by ICM-Pro (further details in Table S3). (b) Structural model of Aβ40 fibrils with 3-fold symmetry (positive stagger) based on solid-state NMR restraints,^89^ showing drug binding pockets 1–13 predicted by ICM-Pro (further details in Table S5). (c–f) Comparison of calculated energies of phenol binding to Aβ40 and tau fibrils. Each point represents an individual compound. Lines of best fit are shown for squared correlation coefficients *R*^2^ > 0.5. Further details are given in Tables S4 and S6.

We performed docking analysis on model structures of Aβ40 (PDB 2LMQ) and tau (PDB 6JQH) fibrils for potential clues as to why the EVOO mixtures and individual phenols were less active against tau than against Aβ40. Using the ICM-Pro docking software, several (>7) putative binding pockets were identified in each structure (Figure 9a,b and Tables S3 and S5). Docking analysis on a selection of EVOO phenolic compounds predicted them to have a preference for 2–3 of these sites in each structure. Interestingly, a strong positive correlation between the calculated total energies for the compounds was observed for binding to the Aβ40 and tau structures (Figure 9c and Tables S4 and S6). The energies were systematically more favorable (*i.e.*, ∼16 kcal/mol lower on average) for phenol binding to Aβ40 than to tau. The contributions of different interactions to the total binding energies revealed poor correlations of the energies for hydrogen bonding (Figure 9d) and hydrophobic interactions (Figure 9e) with the two structures, but a moderate positive correlation existed between the van der Waals energies of binding to Aβ40 and tau (Figure 9f). The limited effects of phenolic compounds on tau may therefore be due to weak van der Waals interactions with the protein. It is noted, however, that these are predicted results on just two specific fibrillar models that may not represent the fibril architectures in our experimental work.

### Implications and Limitations of the Results

This work provides the first experimental verification that phenolic mixtures in EVOO can modify amyloid formation. The results for Aβ should be interpreted with some caveats.

First, we have chosen to focus on Aβ40 rather than Aβ42. The latter isoform is more aggregation-prone and prevalent in AD plaques than the shorter variant and is thought to be more pathogenic.^90^ However, Aβ40 amyloid deposits predominate in the leptomeningeal and cortical arteries of patients affected by cerebral amyloid pathology, which is considered an early step in AD pathogenesis.^91^ Both isoforms are, therefore, important in the search for neuroprotective agents. Many reported *in vitro* screens of inhibitors have used Aβ40 as the representative amyloid-β peptide,^92^ and only a handful of designed synthetic compounds have demonstrated selectivity for Aβ42 over Aβ40.^90^

Second, this work was conducted *in vitro* and naturally does not address the bioavailability of EVOO phenols or whether their antiaggregation effects are reproduced *in vivo*. Many of these compounds are lower in concentration than oleuropein in EVOO, but as bioavailability differs greatly from one phenol to another, the most abundant compounds may not result in the highest concentrations of active metabolites in target tissues.^93^ Indeed, the metabolism of secoiridoid aglycones like oleuropein releases hydroxytyrosol or tyrosol and elenolic acid by enzymatic hydrolysis in the gastrointestinal tract, and circulating oleuropein concentrations may be low.^94^ Ingested dietary phenolic compounds must be absorbed from the gastrointestinal tract, where microbiota can degrade complex polyphenols into low molecular weight phenolics and perform other biotransformations.^95^ Polyphenols in the form of esters, glycosides, or polymers cannot be absorbed directly and must first undergo hydrolysis. Further hepatic transformations of the absorbed phenolic compounds result in a complex distribution of unmodified, fragmented, and partially methylated, sulfated, and glucuronidated compounds.^93^ The circulating phenolics must then cross the BBB, a tightly regulated, selectively permeable endothelial layer, to be effective.^95^ In the case of flavonoids, it was proposed that transmembrane diffusion of phenols across the BBB correlates with their lipophilicity, such that the brain uptake of less polar derivatives such as methylated derivatives is higher than the uptake of more polar (*e.g.*, sulfated) metabolites.^96^ The EVOO flavonoids apigenin^97^ and naringenin^98^ cross the BBB and exert neuroprotection, as do secoiridoids including oleuropein.^99^

The results here provide motivation for further studies to investigate the effects of EVOO phenolic compounds on Aβ40 and tau aggregation *in vivo*, the effects of the various known metabolic transformations on these activities, and how the mixtures are absorbed and metabolized compared to individual EVOO phenols.

## Supporting Information Available

Alternative representation of the reverse-phase HPLC chromatograms (Figure S1); region of the ^1^H COSY spectrum and corresponding1D spectrum (Figure S2); reproduction of ThT analysis of Aβ40 aggregation kinetics on a logarithmic time scale (Figure S3); soluble oligomers of Aβ40 isolated in the supernatant after centrifugation (Figure S4); negative-stainTEM images of tau filaments; computational analysis of phenolic-binding proteins (Figure S5); retention time, linearity range, and regression equation (Table S1); summary of the HPLCanalysis of EVOO compound (Table S2); output from the ICM Pro (Molsoft) pocket finder algorithm for 6QJH (tau) (Table S3) and 2LMQ (Aβ) (Table S5); predicted binding energies (ICM Pro, Molsoft) for compounds docked to the tau filament (PDB 6QJH) (Table S4) and the amyloid beta filament (PDB 2LMQ) (Table S6) (PDF)
