# Supplementary material for: Characterization of Olive Oil Phenolic Extracts and Their Effects on the Aggregation of the Alzheimer’s Amyloid-β Peptide and Tau
Source: ACS Omega. 2024 Jul 17;9(30):32557–78. doi: 10.1021/acsomega.4c01281 (PMC11292642; doi:10.1021/acsomega.4c01281)
Supplement: Supplementary file 1 — ao4c01281_si_001.pdf [file ao4c01281_si_001.pdf]

Supplementary Information for

Characterization of olive oil phenolic extracts and their effects on the aggregation of the Alzheimer's amyloid-β peptide and tau

Bakri Alaziqi,[a,b] Liam Beckitt,[a] David J. Townsend,[a] Jasmine Morgan,[c] Rebecca Price,[d] Alana Maerivoet,[d] Jillian Madine,[d] David Rochester,[a] Geoffrey Akien,[a] & David A. Middleton.*[a]

[a]Department of Chemistry, Lancaster University, Lancaster, LA1 4YB, United Kingdom

[b]Department of Chemistry, University College in Al-Qunfudah, Umm Al-Qura University, 1109 Makkah Al-Mukarramah, Saudi Arabia

[c]Department of Biology, Edge Hill University, Ormskirk, L39 4QP, United Kingdom

[d]Department of Biochemistry, Cell and Systems Biology, Institute of Systems, Molecular and Integrative Biology, University of Liverpool, L69 7ZB, United Kingdom

***Corresponding Author**: d.middleton@lancaster.ac.uk

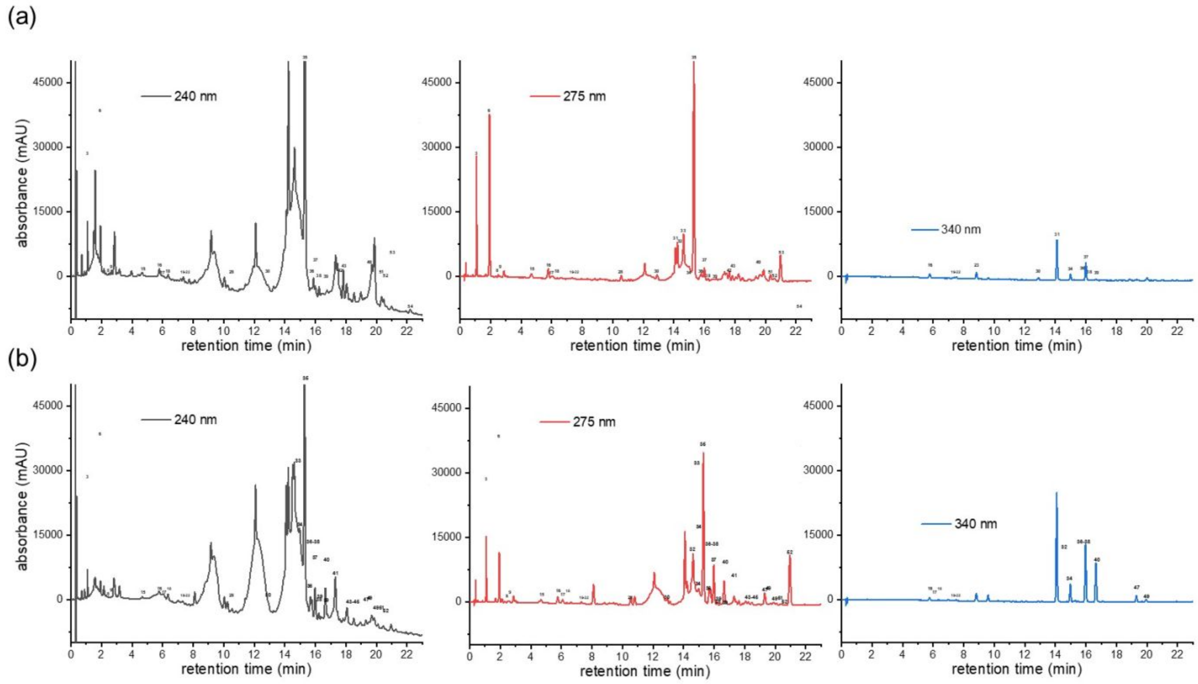

Figure S1. Alternative representation of the reverse-phase HPLC chromatograms from Figure 1b.

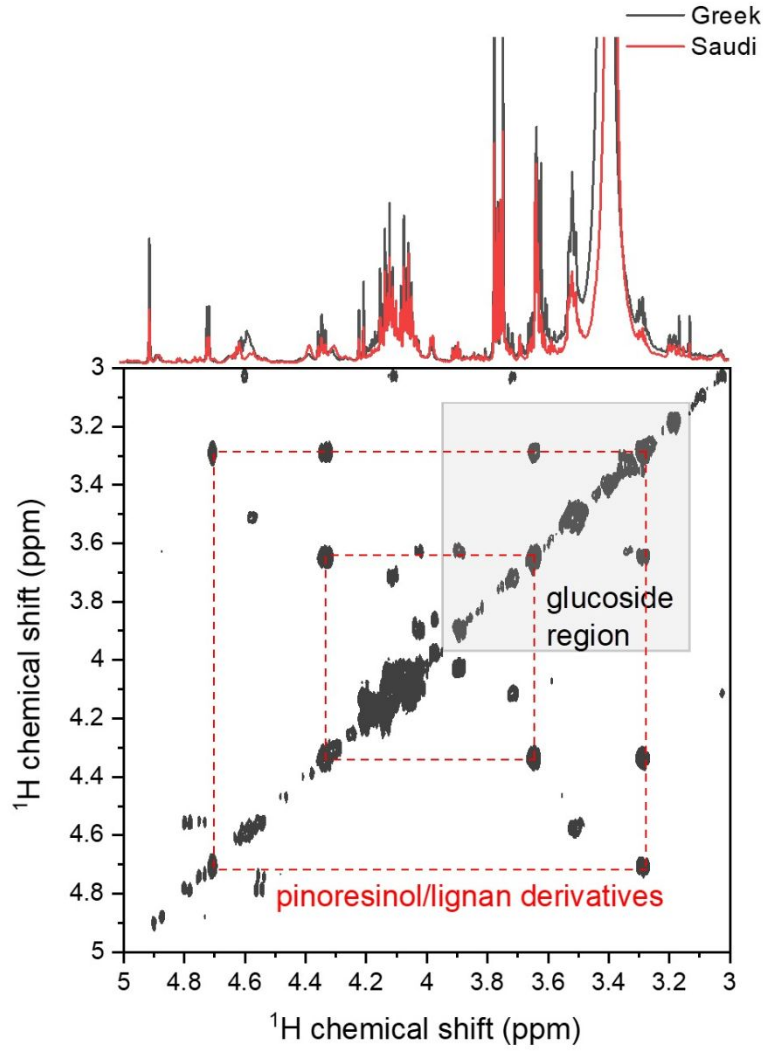

Figure S2. Region of the ^{1}H COSY spectrum of Greek EVOO extract and (top) the corresponding 1D spectrum overlaid with the spectrum of Saudi EVOO extract.

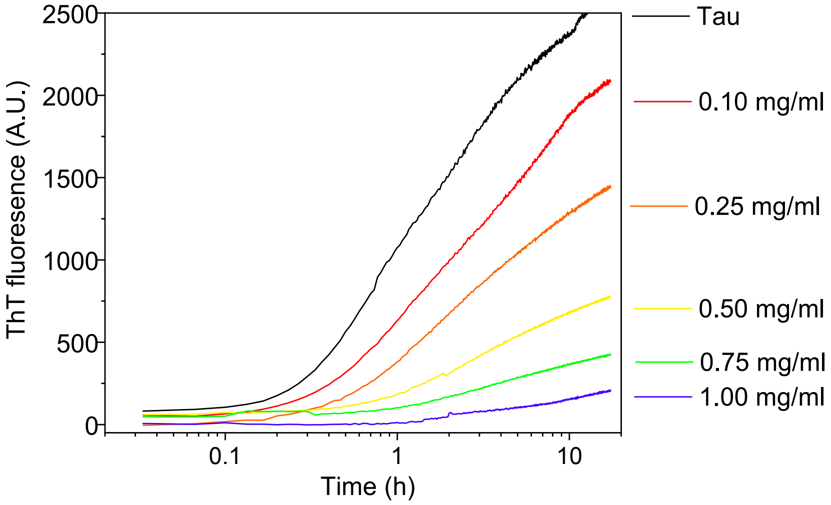

Figure S3. Reproduction of Figure 3 (a) of the main text on a logarithmic time scale.

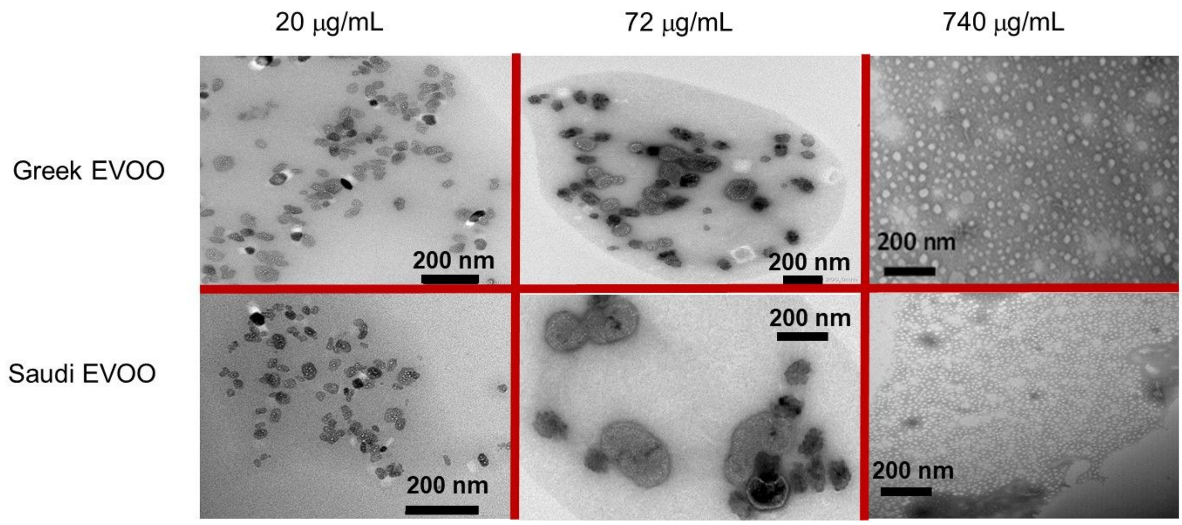

Figure S4. Soluble oligomers of Aβ40 isolated in the supernatant after centrifugation, from pre-formed fibrils treated with EVOO extracts at 3 concentrations.

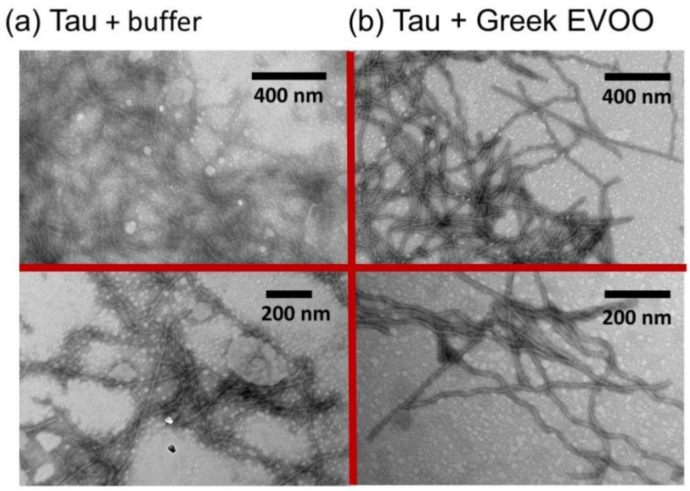

Figure S5. Negative-stain TEM images of tau filaments formed alone after 3 d (a) and after addition of 500 μg/mL Greek EVOO extract (b).

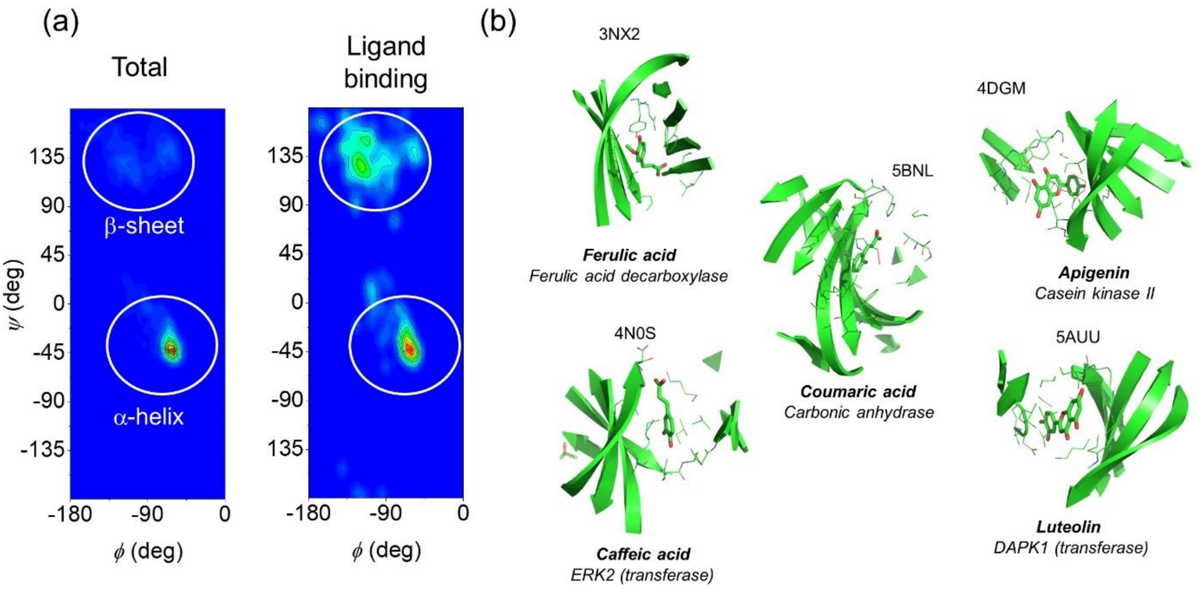

Figure S6. Computational analysis of phenolic-binding proteins. (a) Secondary structure content of unrelated phenolic binding proteins (76 in total) defined according to backbone ϕ and ψ angles. Left: summary of all residues in the proteins. Right: Only residues ≤ 5 Å from one or more sites in the phenol ligand. Structural coordinates were selected of all proteins in the PDB containing the following phenolic compounds: apigenin, caffeic acid, coumaric acid, ferulic acid, luteolin, naringenin, pinoresinol, quercetin, tyrosol, vanillic acid. (b) Examples of phenolic binding sites in selected protein structures from the PDB.

Table S1. Retention time, linearity range and regression equation of detected phenolic compounds in Greek and Saudi EVOOs by HPLC.

Phenolic Compound	Retention time (min)	Linearity range ug/ml	R	R^2	Regression equation
Hydroxytyrosol	1.0	1-30	0.999	0.999	4494.9x – 243.216
Tyrosol	1.9	1-30	0.999	0.999	6562.8x – 352.919
Vanillic acid	2.9	0.05-0.5	0.997	0.994	17900.0x + 307.488
Caffeic acid	3.0	0.05-0.5	0.987	0.974	25926.8x + 222.960
p-Coumaric acid	5.7	0.05-0.5	0.997	0.995	36462.7x + 235.056
Ferulic acid	7.4	0.05-0.5	0.999	0.999	22615.9x – 35.2171
Oleuropein	13.0	1-30	0.999	0.999	918.1x – 132.863
Luteolin	14.1	1-30	0.999	0.999	34492.4x – 4,138.65
(+)-Pinoresinol	14.6	1-30	0.998	0.997	4384.1x – 1,434.26
Naringenin	15.3	1-30	0.999	0.999	22711.6x – 3,923.09
Apigenin	16.0	0.1-20	0.999	0.998	2,442.6x + 3,054.90

R: The coefficient of correlation.

R^2: The coefficient of determination.

Table S2. Summary of the HPLC analysis of EVOO compound binding (20 μg/mL Saudi extract) to Aβ40 fibrils (20 μM monomer equivalent).

Retention time (min)	Compound	Normalized peak intensity[a]		% bound
		-fibril	+fibril	
1.0	Hydroxytyrosol	21.9	21.7	1
1.2	Unknown	7.9	1.6	80
1.7	Unknown	2.0	0.0	100
1.9	Tyrosol	37.1	31.5	15
2.9	Vanillic acid	2.9	0.0	100
3.0	Caffeic acid	2.4	0.0	100
8.1	Unknown	9.6	11.6	0
8.9	Unknown	2.1	0.0	100
9.2	Unknown	65.9	9.7	85
9.4	Unknown	100.0	0.0	100
12.1	Unknown	15.9	15.9	0
14.1	Luteolin	16.1	4.1	74
14.3	Unknown	6.0	5.3	12
14.5	Unknown	24.2	15.4	36
14.6	(+)-Pinoresinol	15.9	13.9	13
15.0	Unknown	4.3	3.4	21
15.3	Naringenin	40.7	41.7	0
15.7	Unknown	5.0	4.4	12
15.8	Unknown	4.5	2.7	39
16.0	Apigenin	13.1	4.7	64
16.7	Unknown	7.5	4.0	47
19.4	Unknown	9.4	0.0	100
21.0	Unknown	31.6	8.5	73
22.6	Unknown	28.1	23.6	16

[a]Normalized to the maximum peak intensity in the absence of fibrils.

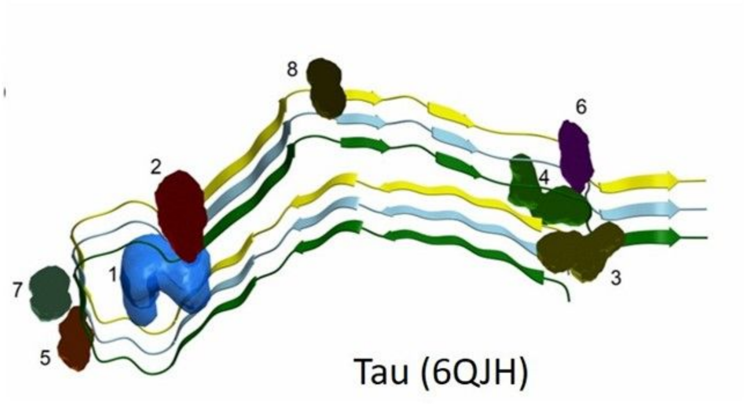

Table S3: Output from the ICM Pro (Molsoft) pocket finder algorithm for 6QJH (tau).

No.	Volume (Å^3)	Hydrophobicity	Buriedness	DLID Merck's Druggable	Radius
1	749	0.656	0.892	1.194	5.634
2	341	0.570	0.793	0.026	4.337
3	191	0.481	0.765	-0.714	3.573
4	131	0.427	0.600	-1.761	3.153
5	130	0.731	0.798	-0.298	3.146
6	114	0.496	0.781	-0.992	3.012
7	110	0.519	0.681	-1.362	2.975
8	106	0.675	0.705	-0.940	2.942

Table S4. Predicted binding energies (ICM Pro, Molsoft) for compounds docked to the tau filament (PDB 6QJH). All values in kcal/mol.

Compound	Energy (kcal/mol)					Binding pocket
	Total	H-bond	Electrost.	H'phobic	VdW	
Hydroxy oleuropein aglycone	-30.9	-3.1	-3.6	-3.9	-52.6	3
Oleocanthal	-30.8	-6.6	-2.9	-2.1	-52.7	1
Hydroxydecarboxy-methyl oleuropein aglycone	-24.9	-2.5	-3.4	-3.3	-50.8	2
Oleuropein aglycone (3,4-DHPEA-EA)	-24.9	-3.0	-1.5	-4.1	-54.6	2
Decarboxymethyl oleuropein aglycone	-24.7	-3.7	-2.6	-1.9	-50.4	2
Hydroxytyrosol acetate (3,4-DHPEA-AC)	-24.6	-1.3	-0.6	-2.2	-41.2	6
Elenolic acid	-22.8	-1.1	0.0	-2.3	-42.8	2
Tyrosol	-20.9	-2.1	-3.2	-1.6	-31.3	4
Coumaric acid	-20.4	-1.6	-1.7	-1.4	-36.8	3
Caffeic acid	-19.9	-2.2	-2.1	-1.7	-36.9	1
Hydroxytyrosol	-19.2	-0.3	-1.9	-1.7	-33.8	1
Cinnamic acid	-19.1	-1.4	-1.3	-1.5	-35.6	2
Apigenin	-11.9	-1.8	-0.2	-3.0	-47.3	2
Vanillic Acid	-5.3	-1.3	-1.5	-1.4	-34.1	2
Luteolin	-3.3	-3.2	-3.2	-1.1	-38.7	2
Ferulic acid	-2.2	-3.4	-5.4	-2.1	-24.5	4
Ouinic Acid	7.0	-8.5	-7.4	0.3	-20.3	2
Tyrosol Glucoside	9.2	-6.9	-6.5	-0.3	-35.0	3
Pinoresinol	27.2	-4.1	-2.5	-2.0	-43.1	2
Gibberellic acid	51.0	-2.9	-1.6	-1.0	-39.9	2

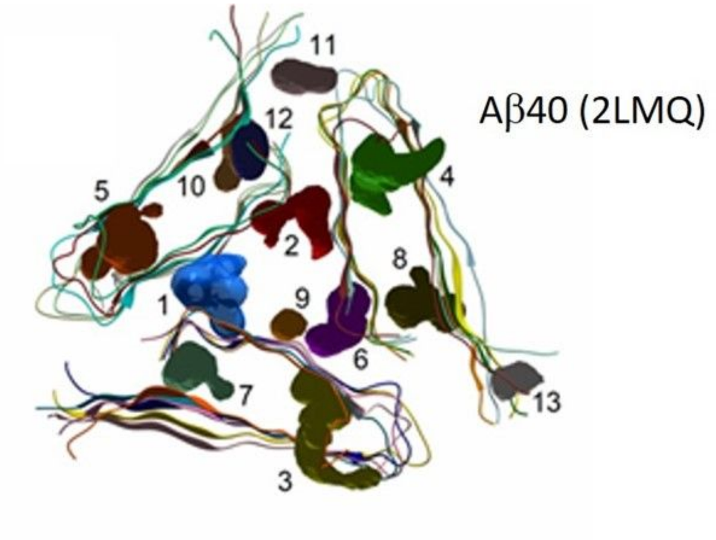

Table S5. Output from the ICM Pro (Molsoft) pocket finder algorithm for 2LMQ (Aβ).

No.	Volume (Å^3)	Hydrophobicity[1]	Buriedness[2]	DLID Merck's Druggable[3]	Radius
1	857	0.618	0.682	0.3793	5.893
2	590	0.741	0.837	0.9928	5. 203
3	571	0.531	0.635	-0.3048	5.147
4	566	0.802	0.885	1. 289	5.132
5	549	0.617	0.761	0.3591	5.082
6	533	0.613	0.743	0. 2576	5.031
7	342	0.615	0.879	0.4711	4.342
8	308	0.541	0.786	-0.1437	4.193
9	301	0.585	0.798	-0.01011	4.161
10	212	0.561	0.596	-1.119	3.703
11	170	0.470	0.715	-1.018	3.442
12	130	0.771	0.985	0.527	3.144
13	104	0.726	0.859	-0. 2306	2.922

[1]Fraction of the pocket surface in contact with hydrophobic protein residues.

[2]Solvent accessible surface of the pocket divided the accessible surface covered by the shell. Values of 0.5 (open) to 1 (buried).

[3]Merck's drug like density score where >0.5 is considered druggable.

[4]Area of pocket / area of perfect sphere. Value of 1 is a sphere.

Table S6. Predicted binding energies (ICM Pro, Molsoft) for compounds docked to the amyloid beta filament (PDB 2LMQ). All values in kcal/mol.

Compound	Energy (kcal/mol)					Binding pocket
	Total	H-bond	Electrost.	H'phobic	VdW	
Hydroxy oleuropein aglycone	-57.1	-4.4	-7.0	-3.5	-73.4	6
Decarboxymethyl oleuropein aglycone	-46.3	-1.7	-3.1	-3.1	-71.4	6
Oleuropein aglycone (3, 4-DHPEA-EA)	-45.0	-0.1	-1.2	-4.9	-81.3	5
Hydroxydecarboxy-methyl oleuropein aglycone	-44.4	-2.7	-4.5	-2.4	-71.7	6
Oleocanthal	-44.2	-2.9	-4.3	-2.8	-65.5	6
hydroxytyrosol acetate (3,4-DHPEA-AC)	-35.7	-1.5	-2.8	-1.8	-50.7	6
Elenolic acid	-31.2	-2.3	-1.9	-1.7	-47.5	6
Tyrosol	-30.6	-4.0	-4.0	-0.5	-39.1	6
Hydroxytyrosol	-29.2	-3.8	-3.5	-0.5	-40.7	6
Caffeic acid	-28.4	-2.2	-5.6	-0.1	-43.9	6
Coumaric acid	-28.2	-2.4	-5.0	-0.2	-42.1	6
Cinnamic acid	-25.2	-0.0	-3.2	-1.9	-41.3	5
Luteolin	-23.8	-0.9	-1.8	-3.5	-60.6	5
Apigenin	-23.6	-1.2	-4.8	-1.3	-57.2	6
Ferulic acid	-20.4	-2.6	-4.0	-0.6	-46.0	6
Vanillic Acid	-17.2	-0.0	-1.8	-1.6	-44.3	5
Tyrosol glucoside	-14.3	-4.6	-3.2	-1.6	-63.4	6
Ouinic Acid	-3.0	-3.8	-6.3	-0.4	-34.3	6
Pinoresinol	1.0	-0.0	-1.2	-5.5	-77.0	5
Gibberellic acid	29.0	-1.5	-3.4	-4.8	-54.4	1
